# The anti-rheumatic drug, leflunomide, synergizes with MEK inhibition to suppress melanoma growth

**DOI:** 10.18632/oncotarget.23378

**Published:** 2017-12-17

**Authors:** Kimberley Hanson, Stephen R. Robinson, Karamallah Al-Yousuf, Adam E. Hendry, Darren W. Sexton, Victoria Sherwood, Grant N. Wheeler

**Affiliations:** ^1^ School of Biological Sciences, University of East Anglia, Norwich Research Park, Norwich, NR4 7TJ, UK; ^2^ School of Pharmacy, University of East Anglia, Norwich Research Park, Norwich, NR4 7TJ, UK; ^3^ Present address: Division of Cancer Sciences, School of Medicine, Ninewells Hospital and Medical School, University of Dundee, Dundee, DD1 9SY, UK; ^4^ Norwich Medical School, University of East Anglia, Norwich Research Park, Norwich, NR4 7TJ, UK; ^5^ Present address: Pharmacy and Biomedical Sciences, Liverpool John Moores University, Liverpool, L3 3AF, UK

**Keywords:** melanoma, leflunomide, selumetinib, MEK inhibitors, combinatorial therapy

## Abstract

Cutaneous melanoma, which develops from the pigment producing cells called melanocytes, is the most deadly form of skin cancer. Unlike the majority of other cancers, the incidence rates of melanoma are still on the rise and the treatment options currently available are being hindered by resistance, limited response rates and adverse toxicity. We have previously shown that an FDA approved drug leflunomide, used for rheumatoid arthritis (RA), also holds potential therapeutic value in treating melanoma especially if used in combination with the mutant BRAF inhibitor, vemurafenib. We have further characterized the function of leflunomide and show that the drug reduces the number of viable cells in both wild-type and *BRAF*^V600E^ mutant melanoma cell lines. Further experiments have revealed leflunomide reduces cell proliferation and causes cells to arrest in G1 of the cell cycle. Cell death assays show leflunomide causes apoptosis at treatment concentrations of 25 and 50 µM. To determine if leflunomide could be used combinatorialy with other anti-melanoma drugs, it was tested in combination with the MEK inhibitor, selumetinib. This combination showed a synergistic effect in the cell lines tested. This drug combination led to an enhanced decrease in tumor size when tested *in vivo* compared to either drug alone, demonstrating its potential as a novel combinatorial therapy for melanoma.

## INTRODUCTION

Melanoma is the most deadly form of skin cancer, causing the majority of skin cancer deaths despite only accounting for 5% of reported skin cancer cases (Skin Cancer Foundation, 2017; [1]) and unlike most other cancers, incidence rates are still on the rise. The cause of melanoma is a combination of exogenous (environmental) and endogenous (genetic) factors [2]. If detected early cutaneous melanomas are easily curable through resection, as unlike many other cancers, they are externally visible and it is only once they have metastasized in later stages that the disease becomes difficult to treat (Skin Cancer Foundation, 2017). Until recently treatment for metastatic melanoma was limited. However, in recent years, a number of new therapies have been developed that provide a better prognosis for patients. These include immunotherapies, in particular immune checkpoint inhibitors such as ipilimumab, pembrolizumab and nivolumab that show remarkable clinical responses in some melanoma patients [3–5]. These therapies however are not without their drawbacks, including immune-related adverse events, limited response rates and possibly also therapy-induced acquired resistance, where modifications to improve the clinical application of these treatments are currently ongoing [6].

Another class of drugs that has been revolutionizing the way in which patients with advanced melanoma are treated is targeted therapies that block oncogenic driver mutations. In particular, targeted therapies that block components of the pro-proliferative mitogen-activated protein kinase (MAPK) pathway such as BRAF (vemurafenib/dabrafenib) and MEK (trametinib/selumetinib). Indeed selective RAF inhibitors have demonstrated clear survival benefit in oncogenic BRAF (predominantly the *BRAF*^V600^ mutation)-driven melanomas (approximately 50% of patients; [7–10]) and results in near-complete abrogation of MAPK signaling in tumors harboring such mutations [11].

The effects of these MAPK treatments are however only transient due to the emergence of a variety of drug resistance mechanisms [12, 13–16] and as a result, metastatic melanoma patients receiving these treatments as monotherapies eventually succumb to their disease. Hence, resistance to such treatments is currently a key issue researchers within the melanoma field are faced with and it is now evident that monotherapy is not the answer. Combinatorial therapy targeting multiple signaling pathways or components within the same pathway is where future strategies lie to try and delay or override tumor resistance, and so provide stronger, more durable responses for patients. A number of drug combinations are currently being investigated in clinical trials with some proving hopeful. Such combinations include combined immunotherapies, BRAF inhibitors in combination with immunotherapies and BRAF inhibitors in combination with MEK inhibitors [17–21].

Leflunomide is an FDA approved drug for the treatment of RA and is an inhibitor of the enzyme dihydroorotate dehydrogenase (DHODH) [22–24], which is the rate limiting enzyme in the *de novo* pyrimidine synthesis pathway. The pyrimidine synthesis pathway consists of six enzymatic reactions, which generate ribonucleotide uridine monophosphate (rUMP). DHODH is located in the inner mitochondrial membrane and catalyzes the conversion of dihydroorotate to orotate, the fourth step of this pathway [25]. Inhibition of DHODH prevents the synthesis of pyrimidines, which has a knock-on effect on the synthesis of pyrimidine derivatives such as the nucleotide bases cytosine and thymine. This ultimately decreases the pool of nucleotides available to make new DNA (as well as RNA). From our previous work carrying out chemical genetic screens on zebrafish and *X. laevis* embryos, leflunomide was shown to have potential therapeutic value in treating melanoma [26]. We further showed that leflunomide inhibits neural crest development by inhibiting transcriptional elongation of genes necessary for neural crest development and also melanoma growth. Genes such as *sox10* and *dct*, which are necessary for normal neural crest and melanocyte development, respectively, exhibited reduced expression [26, 27]. The effect leflunomide has on *Xenopus* and zebrafish embryos is phenotypically similar to the suppressors of Ty 5 and 6 (*spt5/spt6)* mutant in zebrafish embryos. *Spt5/spt6* have been shown to be involved in transcriptional elongation [28]. Our previous work showed that leflunomide reduced cell viability in three melanoma cell lines harboring the *BRAF*^V600E^ mutation [26]. However, it is not known if leflunomide affects melanoma cells that do not harbor *BRAF* mutations and details of how leflunomide exerts its anti-melanoma effects are currently unknown.

In this present study we investigate the action of leflunomide in melanoma cells. We then go on to show that as well as combinatorialy acting with vemurafenib [26], leflunomide synergizes with selumetinib to inhibit melanoma cell growth and decrease tumor size *in vivo*. Taken together our data suggest that leflunomide used in combination with MEK inhibition acts as a potent therapeutic drug combination for the treatment of advanced stage melanoma.

## RESULTS

### Leflunomide decreases the viability of melanoma cells by inducing cell cycle arrest and cell death

In clinical practice, assessment of *BRAF*^V600^ mutation status is the only molecular determinant currently used to influence standard-of-care in melanoma patients. We have selected a panel of eight human melanoma cell lines to further characterize the potential effects of leflunomide as an anti-melanoma drug (Supplementary Table 1), where half habour the *BRAF*^V600E^ mutation and the remainder are wildtype for *BRAF* (*BRAF*^WT^; Supplementary Table 1). As expected *BRAF*^V600^ mutant cells are more sensitive to vemurafenib treatment than *BRAF*^WT^ lines (Supplementary Figure 1 and Table 1).

**Table 1:** Response of melanoma cells to leflunomide, vemurafenib and selumetinib*

Cell type^**^	IC50 (µM) Leflunomide	IC50 (µM) Vemurafenib	IC50 (µM) Selumetinib	BRAF Status	NRAS Status
**M202**	68.1	1.7	0.5	wt	Q61L
**M285**	61.5	n/a	0.5	wt	wt
**M375**	64.1	n/a	0.1	wt	wt
**M296**	111.8	2	0.47	wt	Q61L
**A375**	57.4	0.7	0.19	Homo V600E	wt
**M229**	58.2	0.3	0.2	Homo V600E	wt
**SKmel28**	166.9	0.8	0.43	Homo V600E	wt
**SKmel5**	122.5	0.15	1.01	Het V600E	wt
**Melanocytes**	147.7	nd	n/a	-	-
**HEK293**	48.1	nd	n/a	-	-
**RD1**	84.2	nd	1.05	-	-

^*^IC_50_ values for eight melanoma cell lines, HEK293, RD1 cells and melanocytes are shown for each of the three drugs.

Abbreviations; n/a, not applicable; nd, not determined; wt, wild-type; Homo, homozygous; and Het, heterozygous. Where applicable amino acid substitutions for NRAS and BRAF are indicated.

^**^Additional genetic information of these cells is provided in Supplementary Table 1.

**Figure 1:**
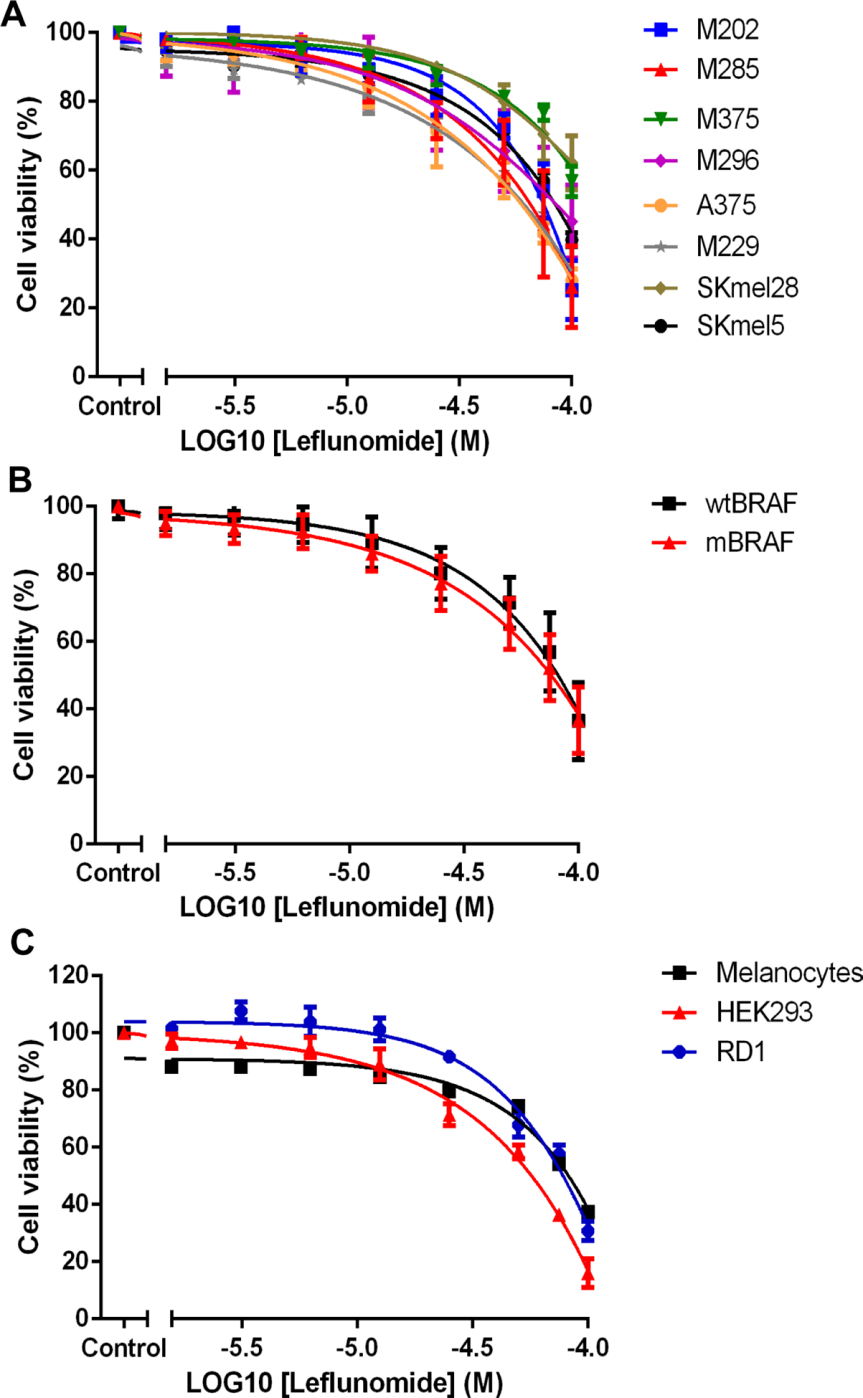
Leflunomide reduces the cell viability of melanoma cell lines **(A**) Leflunomide causes a dose-dependent decrease in cell viability in eight human melanoma cell lines. *BRAF*^WT^ cell lines; M202 (blue), M285 (red), M375 (green) and M296 (purple). *BRAF*^V600E^ mutant cell lines; A375 (orange), M229 (grey), SKmel28 (khaki) and SKmel5 (black). Cell viability was determined by using CellTiter-Glo reagent and all values are represented as a percentage (%) relative to the vehicle control. Data is presented as the mean ± SEM of three independent experiments each performed with cell culture triplicates. (**B**) Leflunomide reduces cell viability at a similar rate in *BRAF*^WT^ (wtBRAF) melanoma cells and *BRAF*^V600E^ mutant (mBRAF) cell lines. The data from the four wildtype cell lines was averaged (black). The same was done for the four *BRAF*^V600E^ mutant lines (red). Cell viability was determined by using CellTiter-Glo reagent and all values are represented as a percentage (%) relative to the vehicle control. Data is presented as the mean ± SEM of twelve independent experiments each performed with cell culture triplicates. (**C**) Leflunomide causes a dose-dependent decrease in cell viability in melanocytes, HEK293 and RD1 cells. Melanocytes (black), HEK293 cells (red) and RD1 cells (blue). Cell viability was determined using CellTiter-Glo reagent and all values are represented as a percentage (%) relative to the vehicle control. Data is presented as the mean ± SEM of three independent experiments each performed with cell culture triplicates.

Cell viability assays using CellTiter-Glo showed that leflunomide reduced the viability of all eight melanoma cell lines in a dose dependent manner (Table 1 and Figure 1A). Both *BRAF*^WT^ and mutant *BRAF* lines were sensitive to leflunomide treatment to comparable levels (Table 1 and Figure 1B). Overall, we observed no obvious differences in leflunomide efficacy based on the mutational status of the melanoma cells (compare Supplementary Table 1 and Table 1). In addition, we analyzed a number of normal human cells and found that they too were sensitive to leflunomide; melanocytes were more resistant than most of the melanoma cells analyzed (Table 1 and Figure 1C).

**Figure 2:**
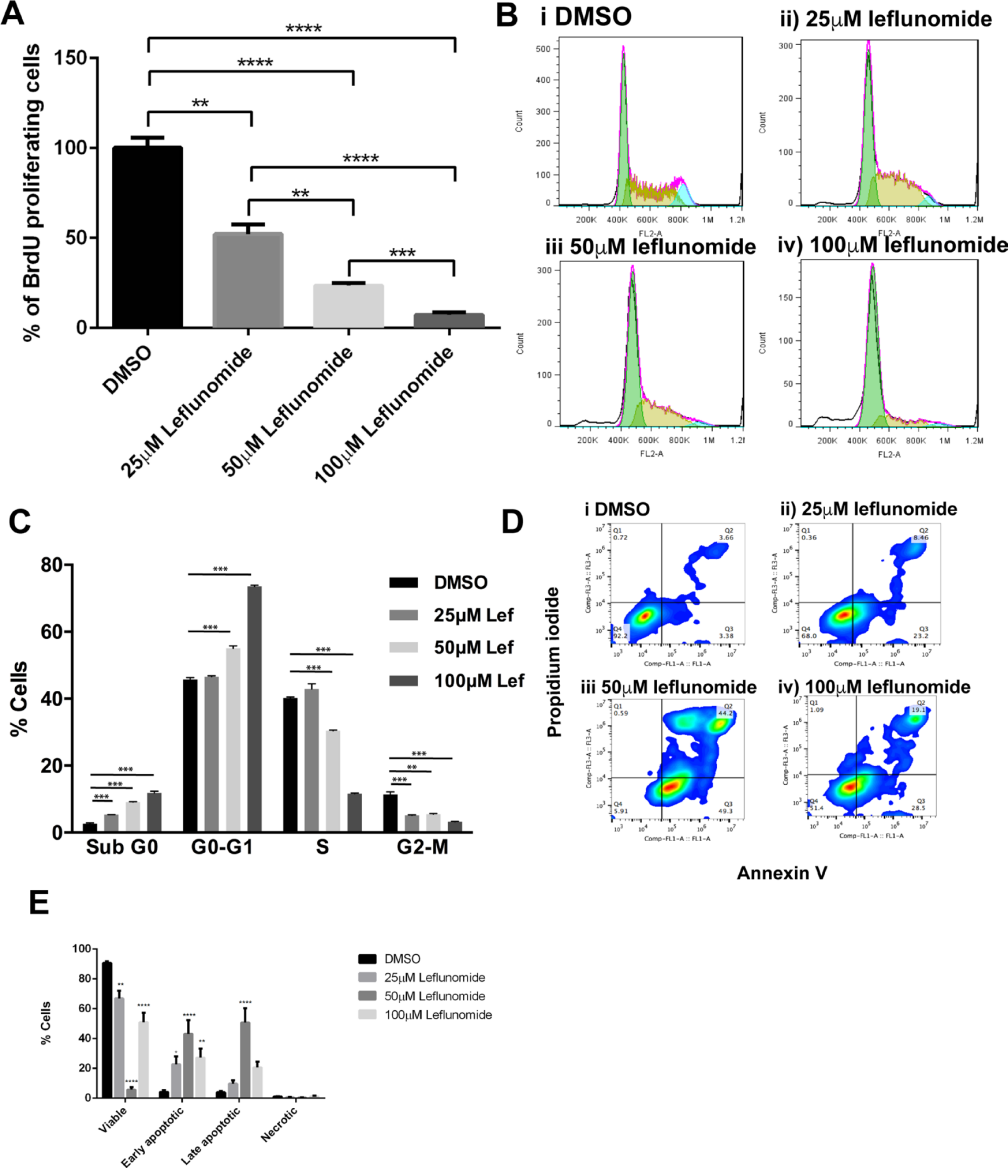
Leflunomide causes a G1 cell cycle arrest in A375 melanoma cells and induces apoptosis **(A**) Leflunomide inhibits cell proliferation in A375 cells. Percentage of BrdU positive A375 cells after 72 hours treatment with leflunomide. Data is presented as the mean ± SEM of the three independent experiments each performed with cell culture triplicates. Asterisks indicate the degree of statistical difference determined by one-way ANOVA with Turkey’s post-hoc test. ^*^P ≤ 0.05, ^**^P ≤ 0.01, ^***^P ≤ 0.001 and ^****^P ≤ 0.0001. (**B**) Representative DNA histogram plots of the cell cycle analysis performed in A375 cells treated for 72 hours with leflunomide. (Bi) shows DMSO treated cells. (Bii), (Biii) and (Biv) show cells treated with 25, 50 and 100 μM leflunomide respectively. (**C**) Leflunomide causes a G1 cell cycle arrest in A375 melanoma cells and induces apoptosis. Cell cycle phase distribution for A375 cells treated for 72 hours with leflunomide. Data is presented as the mean ± SEM of three independent experiments each performed with cell culture triplicates. Asterisks indicate the degree of statistical difference comparing DMSO control to the varying concentrations of Leflunomide using student’s *t*-tests. ^*^P ≤ 0.05, ^**^P ≤ 0.01, ^***^P ≤ 0.001 and ^****^P ≤ 0.0001. (**D**) Representative pseudo plots of cell death analysis determined by flow cytometry. A375 cells were treated with DMSO, 25, 50 and 100 μM leflunomide for 72 hours and stained with annexin V and PI. The numbers indicate the percentage of cells present in each quadrant. (**E**) Graph quantifying the percentage of A375 cells that are viable, early apoptotic, late apoptotic and necrotic after 72 hours of treatment with leflunomide. Data is presented as the mean ± SEM of three independent experiments each performed with cell culture triplicate. Asterisks indicate the degree of statistical difference comparing each leflunomide condition to the DMSO control determined by two-way ANOVA with Turkey’s post-hoc test. ^*^P ≤ 0.05, ^**^P ≤ 0.01, ^***^P ≤ 0.001 and ^****^P ≤ 0.0001.

A375 cells are representative of the panel of melanoma cells in their clear response to leflunomide treatment, which can be easily observed in treated monolayers (Supplementary Figure 2A). To determine why there was a reduction in cell viability upon treatment with leflunomide we first investigated cell proliferation in response to treatment by staining A375 cells with BrdU to determine cell proliferation. The number of BrdU positive cells in each of the treatment conditions showed a clear dose-dependent decrease in the number of proliferating cells in response to increasing concentrations of leflunomide (Figure 2A, Supplementary Figure 2B).

To determine if leflunomide was affecting a particular stage of the cell cycle, analysis was carried out using propidium iodide (PI) to stain for cellular DNA content. A375 cells were stained with PI following a 72-hour treatment with DMSO, 25, 50 or 100 μm leflunomide (Figure 2B). The G0-G1 phase of the cell cycle, increased in a dose-dependent manner in response to leflunomide treatment (Figure 2C). From the DMSO control 45.71% of cells are actively cycling through G1, which increased to 46.56%, 55.05% and 73.56% upon treatment with 25, 50 and 100 μM leflunomide, respectively. In contrast the number of cells in S-phase decreased from 40.26% in DMSO control cells to 42.93% in 25 μM leflunomide treated cells, 30.41% in 50 μM leflunomide treated cells and 11.60% at 100 μM leflunomide (Figure 2C). Thus, with increasing concentrations of leflunomide, the cells become arrested in the G1 phase of the cell cycle and the number of cells in S phase significantly decreases. The percentage of cells in G2-M at 25 µM was reduced by 50% compared to the DMSO control, however the percentage of cells in G2-M for 50 and 100 µM leflunomide does not alter drastically compared to the 25 µM leflunomide. Interestingly, the percentage of cells populated in sub-G1 gradually increased in a dose-dependent manner. In DMSO treated cells 2.60% cells were in sub-G1. This increased to 5.36%, 9.12% and 11.84% upon treatment with 25, 50 and 100 μM leflunomide respectively, suggesting that there could be an increase in the number of cells undergoing apoptosis in response to the drug treatment (Figure 2B, 2C).

To further examine if the cells were undergoing apoptosis we tested their response to leflunomide treatment using Annexin V staining. In control samples the majority of the cells were viable as expected, but treatment with leflunomide led to pro-apoptotic effects, which was most prominent when cells were treated with 50 µM of the drug (Figure 2D(iii)). At 100 µM leflunomide unexpectedly led to an increase in the number of viable cells (from 5.91% up to 51.4%), with concomitant decrease in the number of early apoptotic (from 49.3% to 28.5%) and late apoptotic/necrotic cells (from 44.2% to 19.1%; Figure 2D). Overall, we postulate that leflunomide induces apoptosis in melanoma cells (as opposed to necrosis), which can be easily observed when the data is summarized as the percentage of cells undergoing types of cell death upon treatment with increasing doses of leflunomide (Figure 2E). At 100 µM we are still seeing cell death, but the apoptosis marker (PS exposure) is lost. The cells could be undergoing toxic effects leading to oncosis or necrosis as indicated by the loss of cell density, but absence of PS exposure (Figure 2D).

In mammalian cells, activation of apoptosis is often strongly controlled by mitochondrial activity [33]. Given the pro-apoptotic effects of leflunomide at lower concentrations observed in melanoma cells (Figure 2), we decided to investigate if the drug could also affect mitochondrial activity in these cells. JC-1 staining was conducted as this is commonly used to measure and detect changes in mitochondrial membrane potential and, thus, is a commonly used indicator of healthy cells and a detector of early apoptosis which involves mitochondrial depolaristion (Supplementary Figure 3A). With increasing concentrations of leflunomide the main population appeared to increase in the FL-2 y-axis, which suggests an increase in red fluorescence and a potential increase in mitochondrial membrane potential (ΔψM), albeit this is a relatively subtle effect. However, there is an increase along the FL-1 channel with rising concentrations of leflunomide, suggesting the mitochondria become depolarized in the presence of the drug, which is indicative of a pro-apoptotic state in these cells (Supplementary Figure 3A).

Finally, to see if overall mitochondrial content was affected upon treatment with leflunomide, Mitotracker green dye, which is independent of ΔψM, was used to assess mitochondrial mass (Supplementary Figure 3B). At 100 μM of leflunomide treatment, there was a substantial increase in the intensity of green fluorescence, suggesting an increase in mitochondrial mass at this drug concentration in the melanoma cells. Indeed, there was a 3-fold increase in the amount of green fluorescence at 100 μM leflunomide when compared to the DMSO control, which was not observed at lower leflunomide concentrations (Supplementary Figure 3C). This, however, is not the correct interpretation. Keij *et al.* (2000) reported that mitochondrial swelling (oncosis) can lead to increased fluorescence signals, since the normally densely-packed self-quenched probes are capable of releasing more photons in the swollen mitochondrion. Thus, at 100 μM leflunomide we are observing mitotoxicity through loss of mitochondrial volume control. This is consistent with oncotic cell death which leads to the loss of cell density in these samples; it was noted that there was a 3-fold increase in the time it took for the 100 μM of leflunomide treatment sample to reach 5000 events for counting by flow cytometry relative to the 25 μM and 50 μM treated sample sets (Supplementary Figure 3D). Loss of ion flux control also explains the loss of the apoptotic marker (PS exposure) at the 100 µM dose (Figure 2D). Overall, we conclude that the differences seen at 100 µM are potentially due to mitochondrial oncosis, which is often linked to a complete overdose of a toxin and is associated with a loss of cells, but at lower concentrations (25 μM and 50 μM) leflunomide induces apoptosis in melanoma cells.

### Investigating the possibility of using leflunomide in combination with a MEK inhibitor to treat melanoma

In recent years it has become widely accepted that combinatorial therapy is a better approach for treating cancer. Within the skin cancer field, there is substantial clinical data supporting MEK inhibitors being used for the treatment of melanoma [34]. Indeed the standard-of-care for BRAF-targeting in melanoma has now predominantly shifted from the use of single BRAF inhibitors to use in combination with MEK inhibitors. Taking this into account, the possibility of using leflunomide in combination with the MEK inhibitor, selumetinib was investigated. The rationale for this is that melanomas are addicted to MEK for proliferation and survival. Therefore inhibition of MEK might reduce survival signaling and sensitize cells to the cytotoxic effects of leflunomide.

**Figure 3:**
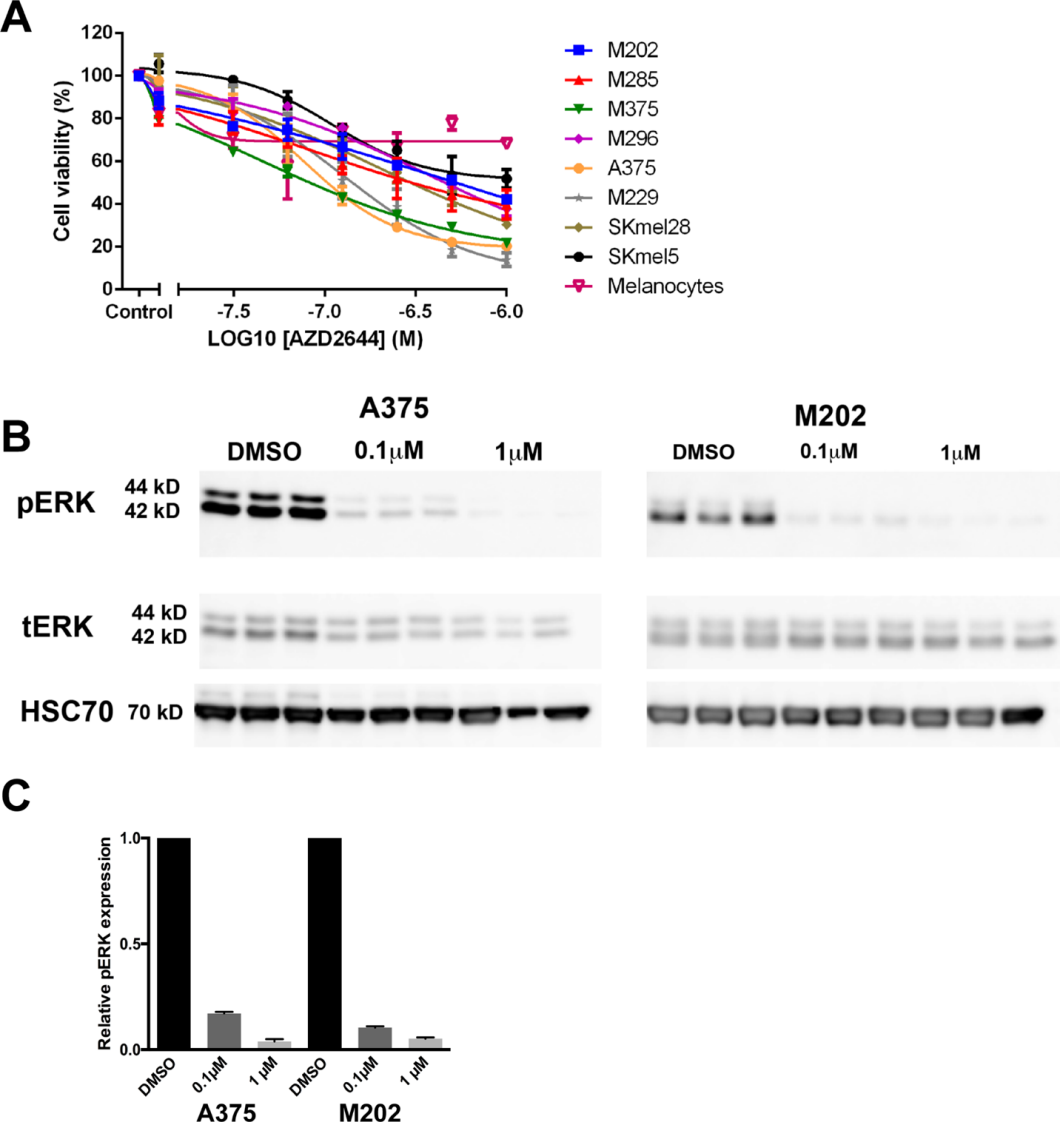
MEK inhibition reduces the viability of melanoma cells (**A**) Selumetinib caused a dose-dependent decrease in cell viability in eight human melanoma cell lines. Melanoma cell lines include M202 (blue), M285 (red), M375 (green) and M296 (purple), A375 (orange), M229 (grey), SKmel28 (khaki), SKmel5 (black) and melanocytes (pink; open triangle). Cell viability was determined by using CellTiter-Glo reagent and all values are represented as a percentage (%) relative to the vehicle control. Data is presented as the mean ± SEM of three independent experiments each performed in triplicate. (**B**) Western blot analysis confirming the decrease in phospho-ERK upon treatment with 0.1 or 1 µM selumetinib in A375 and M202 melanoma cell lines in triplicate. The molecular weights are shown on the left. Results for pERK and total ERK (tERK) are from a single experiment representative of three independent experiments. (**C**) Quantification data from Western blot.

Selumetinib (AZD2644) treated cell viability assays were carried out using CellTiter-Glo on all eight of the melanoma cell lines (Table 1 and Figure 3A). A dose-dependent decrease can be seen in the number of viable cells upon 72-hours treatment with selumetinib in all eight of the melanoma cell lines (Figure 3A). There was a broad range of variation in the level of sensitivity to selumetinib, which is similar to the variation observed for leflunomide (Table 1 and Figure 1A). For example the most sensitive melanoma cell line to selumetinib was M375 with an IC_50_ of 0.10 µM, whereas the least sensitive melanoma cell line was SKmel5 with an IC_50_ of 1.01 μM (Table 1 and Figure 3A).

Non-melanoma cells including melanocytes were also treated with selumetinib (Figure 3A and Supplementary Figure 4) and their sensitivity to the drug determined (Table 1). Interestingly, all three non-melanoma cell types were less sensitive to selumetinib compared to the eight melanoma cell lines. The RD1 cell line was the most sensitive with cell viability being reduced to 55% at 1 μM selumetinib (Table 1 and Supplementary Figure 4). This sensitivity was very close to the least sensitive of the melanoma lines, SKmel5, where its cell viability was reduced to just 51.80% at the same concentration (Table 1). Overall, these findings are in support of previous work showing that melanoma cells are more sensitive to MEK inhibition than normal cells, with drug sensitivity to selumetinib in the same range as detected here (as previously reviewed [35]).

To confirm selumetinib was active and acting ‘on-target’ as a MEK inhibitor, western blots were performed to detect the levels of phospho-ERK (pERK) on treated A375 and M202 cells. As ERK is a direct substrate of MEK a decrease in pERK would be anticipated in response to selumetinib treatment. It can be clearly seen that the amount of pERK protein decreases in a dose-dependent manner in melanoma cells in response to selumetinib treatment (Figure 3B and 3C). Overall, this confirms that selumetinib is effectively inhibiting its target and can reduce cell viability in melanoma cells.

### Leflunomide and selumetinib exhibit synergistic activity in human melanoma cells

**Figure 4:**
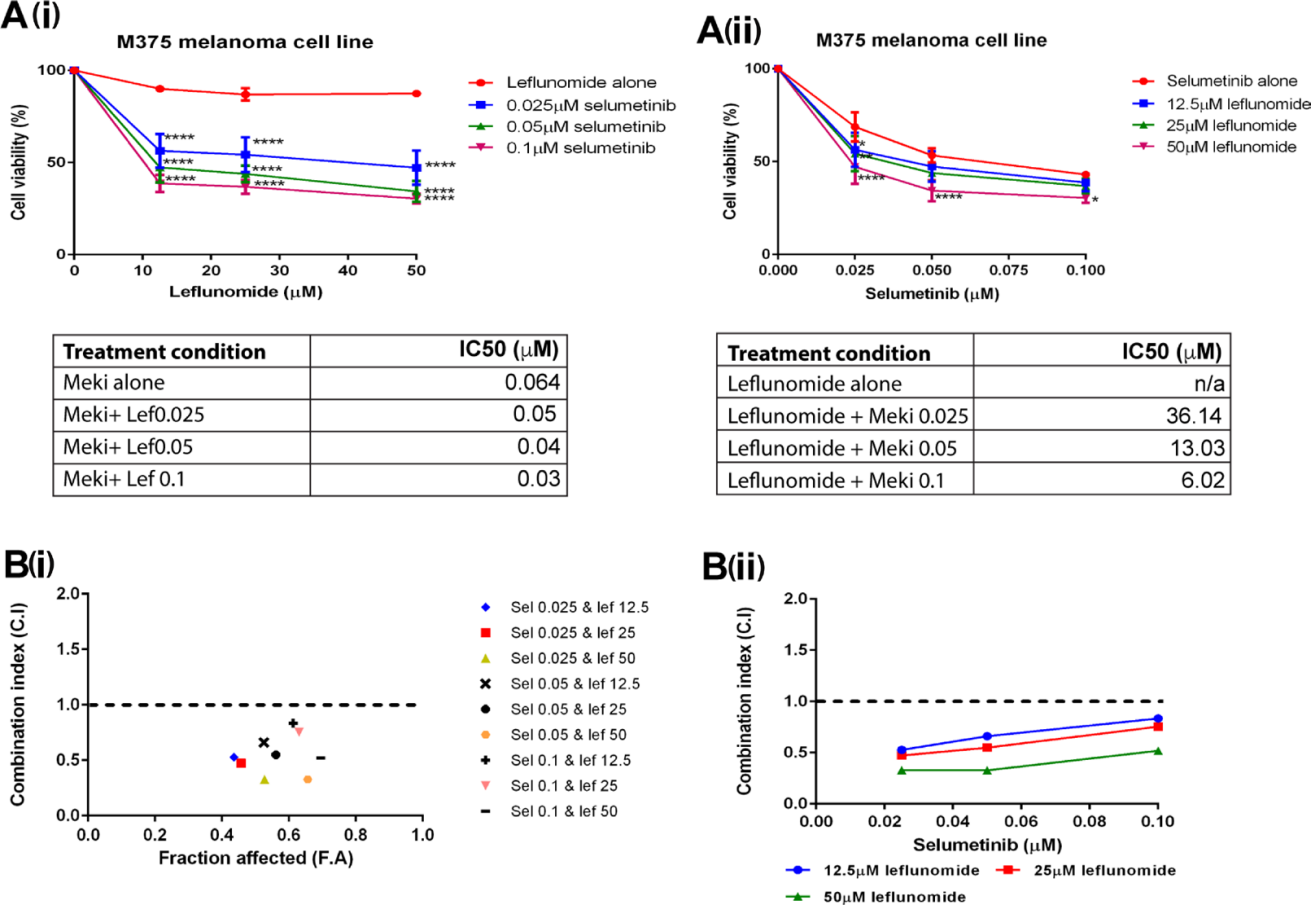
Leflunomide and Selumetinib synergize in melanoma cells Cell viability plots when different concentrations of Selumetinib and Leflunomide are added to the cells simultaneously. Graph **A(i)** shows he concentrations of leflunomide along the x-axis. The statistical analysis on this graph compared the combinations of drugs to leflunomide alone. Graph **A(ii)** shows the concentrations of selumetinib along the x-axis. The statistical analysis on this graph compared the drug combinations to selumetinib alone. All values are represented as a percentage (%) relative to the vehicle control. Data is presented as the mean ± SEM of three independent experiments each performed in triplicate. Asterisks indicate the degree of statistical difference comparing each leflunomide and selumetinib condition to leflunomide alone (graph A(i)) or selumetinib alone (graph A(ii)). Statistical analysis was determined by two-way ANOVA with Turkey’s post-hoc test. ^*^P ≤ 0.05, ^**^P ≤ 0.01, and ^****^P ≤ 0.0001. (A) Combination index values for M375 melanoma cell line with leflunomide and selumetinib in combination at increasing concentrations. **B(i)** Along the x-axis is the Fraction Affected (FA) which corresponds to the cell viability data inputted (i.e. what fraction of the cells were affected/how much of the cell viability was being reduced by this combination of leflunomide and selumetinib). Along the y-axis is the CI values. A dotted line placed across the CI value of 1 makes it easier to see if a particular combination of leflunomide or selumetinib was synergistic or not. A CI value of 1 suggests that drug combination is acting additively. A value greater that 1 suggests the drug combination is acting antagonistically and a value below 1 suggests they are working synergistically. The closer the value to 0 the stronger the synergism. **B(ii)** This graph utilises the CI value data with the CI values again shown along the y–axis but along the x-axis is the concentration of selumetinib with the data sets on the graph corresponding to the leflunomide concentrations. The degree of synergism increases with increasing concentrations of leflunomide. The Synergy graphs for the other melanoma lines tested are shown in Supplementary Figure 5.

The preceding results showed both leflunomide (Figure 1) and selumetinib (Figure 3) were effective at reducing cell viability in the melanoma lines. Prompted by these results, experiments were designed to determine if the combination of leflunomide and selumetinib could reduce cell viability further than either drug alone. Combinatorial cell viability assays were carried out on all eight of the melanoma lines. For these cell viability assays the concentrations of leflunomide used were 12.5, 25 and 50 μM and for selumetinib the concentrations used were 0.025, 0.05 and 0.1 μM. Two cell viability graphs were generated for each melanoma line (Figure 4A and Supplementary Figure 5). This was done in order to complete statistical analysis comparing the drug combinations to each drug alone. The statistics shown on these graphs determined that the drug combinations were significantly better at killing melanoma cells than either drug alone. All of the eight melanoma cell lines responded to the combinations of leflunomide and selumetinib. The most statistically significant combination of leflunomide and selumetinib was at 50 μM leflunomide and 0.1 μM selumetinib (a ratio of 500:1). Therefore this specific combination of leflunomide and selumetinib indicates that these concentrations could be within the optimal working concentration range for this drug combination in melanoma cells.

The next question was are the drugs acting synergistically or not? One approach of determining drug synergy is by calculating combination index (CI) values for multiple drug combinations using the Chou and Talalay method [32]. CI values were calculated for each separate combination of leflunomide and selumetinib (non-constant ratio). This was done for all eight of the melanoma cell lines. For each cell line, two graphs were plotted to demonstrate the synergism of the two drugs as shown in Figure 4B(i) and 4B(ii). For the majority of the lines tested, high dose leflunomide (50 μM) showed synergistic effects when used in combination with selumetinib (Table 2).

**Table 2:** Summary CI values for all melanoma cell panel*

	Leflunomide 12.5 µM			Leflunomide 25 µM			Leflunomide 50 µM		
Melanoma cell line	MEKi 0.025 µM	MEKi 0.05 µM	MEKi 0.1 µM	MEKi 0.025 µM	MEKi 0.05 µM	MEKi 0.1 µM	MEKi 0.025 µM	MEKi 0.05 µM	MEKi 0.1 µM
**A375**	1.621	1.526	1.561	1.59	1.699	1.814	1.414	1.507	1.646
**M375**	0.528	0.661	0.834	0.473	0.549	0.754	0.328	0.327	0.519
**M296**	0.837	1.103	1.479	1.249	1.13	1.381	0.897	0.703	0.655
**M202**	1.059	1.182	1.42	1.1	1.013	1.13	0.947	0.532	0.603
**M229**	0.827	0.743	0.939	1.011	0.831	0.94	0.801	0.617	0.846
**M285**	0.704	0.95	1.033	0.657	0.709	0.777	0.568	0.665	0.515
**SKMEL28**	0.536	0.699	1.279	0.812	0.841	1.444	1.031	0.85	1.058
**SKMEL 5**	1.397	1.393	0.3	1.301	1.445	0.237	1.381	1.152	0.112

^*^Purple indicates antagonism, orange indicates additive and green indicates synergism.

It was further investigated whether addition of Leflunomide or Selumetinib alone 24 hours before addition of the other drug had any effect on their synergy. We selected four lines; one that showed obligate antagonism to the drug combination treatment (A375) to see if drug interactions could be improved, one that showed obligate drug synergy (M375) to check if the desired drug interactions might be lost with a different dosing regime and two lines that showed mostly synergistic, but also some additive response (M229, and M285; i.e. borderline drug synergy response) to treatment (Table 2).Supplementary Tables 2–5 show the CI values for these treatments of the four melanoma lines. For A375 cells there was a slight improvement when the drugs were added incrementally, but this was only at one concentration and the synergy effect was mild (Supplementary Table 2). For M229 and M375 cells, incremental drug addition led to loss of drug synergy at most of the concentrations tested (Supplementary Table 3 and Supplementary Table 4). M285 on the other hand did show drug synergy for every concentration tested when the drugs were added one after the other, but this represented only a mild improvement to when the drugs were added simultaneously (Supplementary Table 5). Overall, these findings suggest that there is likely to be a greater chance of achieving drug synergy in melanoma cells when leflunomide and selumetinib are co-administered at the same time.

Finally, we investigated if the drug combination could increase apoptosis in melanoma cells with respect to monotherapy by analyzing PARP1 cleavage (cPARP), which is a sensitive marker of cells undergoing apoptosis. cPARP is increased in melanoma cells in response to the drug combination, with concomitant decrease in levels of the anti-apoptotic Bcl-2 family member, MCL-1 (Supplementary Figure 6), showing that combined leflunomide and selumetinib treatment increases apoptosis in melanoma cells compared with individual drug treatment. Selumetinib treatment however results in higher levels of the BH3-only pro-apoptotic protein, BIM, than the drug combination (Supplementary Figure 6). These findings are more difficult to interpret as aside from the pro-apoptotic activity of BIM in inducing BAX/BAK oligomerization on mitochondria to release cytochrome c and induce intrinsic apoptosis, BIM has also been shown to possess pro-survival effects in cancer cells [36]. Overall, we conclude that combined leflunomide and selumetinib can increase apoptosis in melanoma cells to a higher level than individual drug treatment alone.

### Leflunomide and selumetinib combine to repress tumor growth *in vivo*

Because the combination of leflunomide and selumetinib showed synergistic activity in a range of melanoma cell lines in our *in vitro* experiments (Figure 4, Table 2, Supplementary Tables 2–5 and Supplementary Figure 5), we wanted to investigate whether the drug combination could also show improved efficacy *in vivo* compared to monotherapy treatment. The M375 cell line showed synergistic activity of the drug combination at all concentrations tested (Figure 4 and Table 2), so we investigated if these cells could be easily engrafted in immunodeficient mice as compared to other human melanoma cells, for *in vivo* studies. Using SCID mice, we developed an engraftment protocol for these cells and compared them to other melanoma lines known to engraft in immunocompromised mice, to ensure palpable M375 tumors could develop in a relatively rapid time-frame (Supplementary Figure 7).

**Figure 5:**
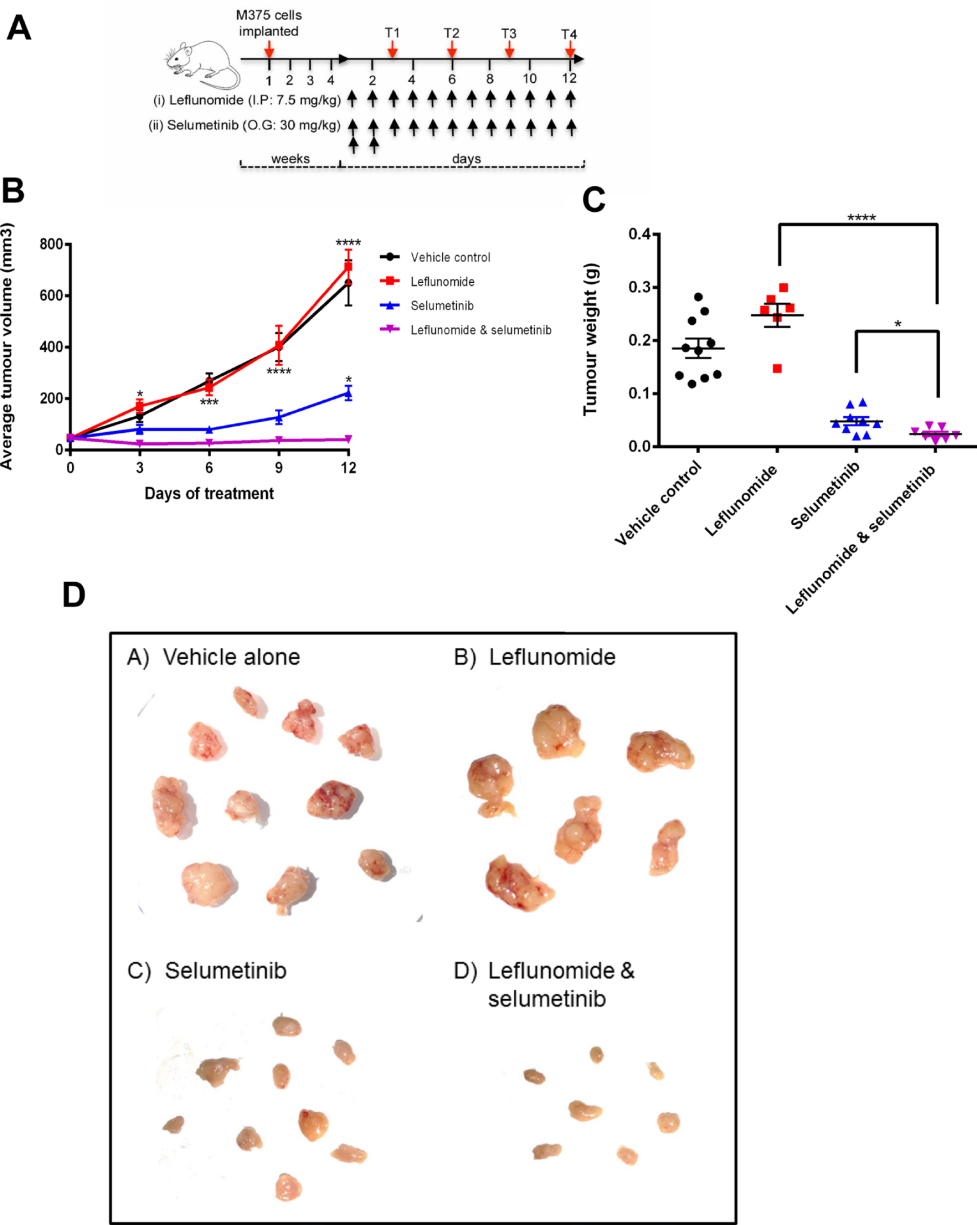
The combination of Leflunomide and Selumetinib reduces tumor growth *in vivo* (**A**) SCID mice xenotransplanted with human melanoma cells (M375; 3 × 10^6^/animal, subcutaneous injection) until tumors were palpable (4-weeks post implantation), were treated daily with leflunomide/selumetinib as individual drugs or in combination as indicated for 12 days. A control arm was also included where comparable vehicle only treatments were administered. Tumor volumes were measured at 4 time-points (T1–T4) during the 12-day treatment period as indicated. I.P., intraperitoneal. O.G., oral gavage. (**B**) The combination of leflunomide and selumetinib reduced the average tumor volume greater than either drug alone. Data is presented as the mean ± SD of one independent experiment. Statistical analysis compares either drug alone to them in combination determined by two-way ANOVA with Turkey’s post-hoc test. ^*^P ≤ 0.05, ^**^P ≤ 0.01, ^***^P ≤ 0.001 and ^****^P ≤ 0.0001. (**C**) The combination of leflunomide and selumetinib reduced tumor weight greater than either drug alone. Data is presented as the mean ± SD of one independent experiment. Asterisks indicate the degree of statistical difference comparing the combination of leflunomide and selumetinib to each drug alone determined by unpaired student *t*-test. ^*^P ≤ 0.05, ^**^P ≤ 0.01, ^***^P ≤ 0.001 and ^****^P ≤ 0.0001. (**D**) Visualisation of the excised tumors from the xenograft study.

Following 4-weeks of tumor growth, drugs were administered in 4 treatment groups; vehicle alone, leflunomide alone, selumetinib alone and leflunomide and selumetinib in combination (10 animals/group) with a daily treatment regime as shown in Figure 5A. In the vehicle control arm, the average tumor volume increased from 46 mm^3^ on day 0 to 650 mm^3^ on day 12, indicating a steady increase in tumor growth over the course of the experiment (Figure 5B). Unexpectedly, leflunomide treatment alone did not reduce tumor volume when compared to the vehicle control. In contrast, selumetinib treatment significantly reduced the average tumor volume, albeit the tumors did continue to grow during the duration of the experiment. Interestingly however, when leflunomide and selumetinib was administered in combination, the tumor volume not only decreased to levels significantly smaller than either drug treatment alone, but importantly tumor growth was suppressed, with tumor volumes remaining steady at the same size over the 12-day treatment period (Figure 5B).

The tumors from the sacrificed mice at the end of the experiment were excised, weighed (Figure 5C) and imaged (Figure 5D). Overall, there is a remarkable drop in tumor size and weights when leflunomide and selumetinib were used in combination. The combination of leflunomide and selumetinib on the effect on the tumor weights was significantly better than either of the two drugs alone (Figure 5C). Taken together, our data show that combination of leflunomide and selumetinib has the capability of not only reducing tumor volume, but also preventing tumor growth *in vivo*.

## DISCUSSION

In recent years the development of new therapies for melanoma has led to a revolution in treatment, which has reflected decades of basic research into the genomic landscape and fundamental immune system behaviour of the disease. The advent of BRAF and MEK inhibitors used in combination has become a standard therapeutic approach in patients with *BRAF*-mutated melanoma. In addition, immunotherapies such as anti-PD-1 antibodies have been effective. In many cases, particularly with small molecule treatments as monotherapies the main problem has been the development of tumor resistance. Therefore, it is important to develop new combination therapies and to identify novel drugs that can be added to the arsenal of anti-melanoma therapies available for patients.

Leflunomide is an immunosuppressive drug which was approved by the FDA in 1998 for the treatment of RA. It has also been shown to inhibit the growth of a number of different cell types including human myeloma cells [37], mitogen-stimulated T-lymphocytes [38], normal human mast cells [39], prostate cancer cells [40] neuroblastoma cells [41] and melanoma cells [26]. In these different cell types the optimum concentration of leflunomide varies. For instance Zhu *et al.* [41] use 100 µM as an optimal concentration for their cell cycle analysis and apoptosis studies. Here we have determined 50 µM to be the optimal concentration to use *in vitro* for apoptosis induction. At higher concentrations we detect potentially off target toxic effects and the cells undergo oncosis. Baumann *et al.* [37] have themselves noted that leflunomide may act independently of DHODH at higher concentrations. That is not to preclude higher doses from therapeutic consideration. Lytic modes of cell death are proinflammatory and in solid tumors, where several mechanisms exist to downregulate innate immune responses and consequent adaptive immune responses, targeted cytotoxicity may facilitate immune activation. In melanoma cells the mechanism of leflunomide action is to inhibit transcriptional elongation [26] which leads to a decrease in cell proliferation.

We have previously identified leflunomide as having therapeutic value in treating melanoma in a mouse xenograft model both on its own and in combination with a BRAF inhibitor [26]. We have now expanded upon these initial studies to show that leflunomide affects the growth of both *BRAF*^WT^ and mutant melanoma cells (Figure 1). This potential allows for leflunomide to be used in all melanoma cases, not just for tumors harboring *BRAF* mutations. We show that at intermediate dose concentrations, leflunomide inhibits G1 arrest and induces apoptosis (Figure 2). To the best of our knowledge, this is the first time the mechanism of action of leflunomide has been investigated in melanoma cells. Finally we also show that leflunomide can act in combination with the MEK inhibitor, selumetinib (Figure 3), to inhibit melanoma growth (Figure 4 and Figure 5).

To determine if the synergism observed *in vitro* between leflunomide and selumetinib had a similar effect *in vivo*, a mouse xenograft study was carried out. This study used the M375 cell line for a drug treatment duration of 12 days. What was obvious from the results of this study was that selumetinib was the more effective drug compared to leflunomide (Figure 5). In this experiment leflunomide alone did not reduce the tumor volume or weight compared to the vehicle control. It is possible that the dose of leflunomide used in this experiment may have been on the border of efficacy on its own. However, the cells were strongly sensitized by leflunomide treatment to the anti-melanoma effect of the MEK inhibitor. Although from this study it cannot be said that the drug synergy observed *in vitro* for M375 translates *in vivo*, what can be stated is that the combination of leflunomide and selumetinib significantly decreased the growth of melanoma *in vitro* and *in vivo* compared to using either drug alone.

Recently leflunomide has been shown to work in combination with doxorubicin to inhibit growth of triple-negative breast cancer [42]. The mechanism the authors suggest is based on their finding that pyrimidine synthesis increases in response to genotoxic stress. Thus, in our results selumetinib could be inducing this response of increased pyrimidine synthesis which in turn is inhibited by leflunomide, thus making the cells less likely to survive. This does not answer why leflunomide has an effect on its own, but does highlight that it could make a potent contribution to combinatorial treatments of malignancies. Whilst future clinical studies are needed to investigate this intriguing possibility further, our findings do highlight some important discoveries about the leflunomide/selumetinib drug combination for such future work. Firstly, the combination shows drug synergy in both *BRAF*^MUT^ and *BRAF*^WT^ melanoma cells (Table 2, Figure 4 and Supplementary Figure 5), suggesting it could be a potent anti-melanoma treatment for patients regardless of genotype. Furthermore, we found no patterns with other common melanoma mutations (Supplementary Table 1) that dictated response rates of the cell lines tested to the combination. Secondly, dosing of the combination has an improved chance of yielding potent synergistic anti-melanoma effects, when administered simultaneously to the cell panel tested (Supplementary Tables 2–5), suggesting designing future dosing regimens where both drugs are administered at the same time. Lastly our pre-clinical *in vivo* model not only demonstrated the potent efficacy of the drug combination at blocking tumor growth (Figure 5), but also highlighted that the therapy is tolerable, as no overt toxicities were detected in the mice during the treatment period.

In conclusion we have shown leflunomide to work in combination both with BRAF and MEK inhibitors in preventing melanoma growth. Future work will determine the mechanism of drug synergy afforded by combined treatment of melanoma cells with leflunomide and selumetinib. Furthermore, additional pre-clinical experiments are needed to determine if melanoma cells can acquire resistance to leflunomide and whether the drug could also be successfully used in combination with anti-melanoma immunotherapies.

## MATERIALS AND METHODS

### Compounds

Vemurafenib (ChemieTek) was dissolved in dimethyl sulphoxide (DMSO; Sigma-Aldrich) and stored at –20° C at stocks of 100 mM. Leflunomide (Sigma-Aldrich) was dissolved in DMSO and stored at 4° C at stocks of 10 mM. AZD6244 (selumetinib; SelleckChem) was dissolved in DMSO and stored at –20° C at stocks of 2 mM. When aliquots of the stock were in use they were stored at 4° C for no longer than two weeks.

### Cell lines and culture

The human melanoma M285, M375 and M296, cell lines were a kind gift from Antoni Ribas (University of California, Los Angeles), and the M202, A375, M229, SKmel28 and SKmel5 cells were a kind gift from Randall T. Moon and Andy J. Chien (University of Washington, Seattle). Primary human melanocytes adult (HEMa-LP) were obtained from Gibco. Human embryonic kidney cells (HEK-293) and rhabdomyosarcoma cells (RD-1) were obtained from the Biomedical Research Centre (University of East Anglia, UK). Human melanoma cells were cultured as previously described [29]. HEMa-LP cells were cultured in Medium-254 (Gibco) with the addition of PMA-Free Human Melanocyte Growth Supplement-2 (HMGS-2; Gibco). HEK-293 cells were cultured in Dulbecco’s modified Eagle medium (DMEM) + GlutMAX (Gibco) supplemented with 10% FBS, 1% L-glutamine and penicillin and streptomycin. RD-1 cells were cultured in Dulbecco’s modified Eagle medium (DMEM) + GlutMAX (Gibco) supplemented with 10% FBS and penicillin and streptomycin. All cells were maintained at 37° C in a 5% CO_2_ air-humidified incubator, were routinely screened for mycoplasma and not cultured beyond passage 25.

### Cell viability assays

Cells that had been seeded 24 hours earlier on poly-L-lysine coated 96-well plates (Sigma-Aldrich) to subconfluency, were treated with drugs at the indicated concentrations for 72 hours. Cytochalasin D (Sigma Aldrich) was used as a positive control. All conditions were repeated in triplicate. Cell viability was determined on day 5 using the CellTiter-Glo Luminescence assay (Promega), according to the manufacturer’s instructions. Luminescence from the plate was read on a BMG LabTech Omega Series plate reader (data analyzed using OMEGA software). Cell viability was calculated as a percentage of the mean vehicle control.

### 5-Bromo-2′-deoxyuridine (BrdU) proliferation assay

A375 melanoma cells were seeded in 12-well plates at a density of 10,000 cells and grown on gelatin-coated coverslips. After 24 hours leflunomide was added to cells at 12.5, 25 or 50 μM (or a vehicle control) for 72 hours. Cells were pulsed for 2 hours with BrdU (Sigma-Aldrich) at a final working concentration of 10 μM. Cells were permeabilised in 2N-HCL + 0.5% Triton X-100. Primary BrdU antibody diluted 1:100 in 1% goat serum was applied and incubated overnight at 4°C followed by Alexa Fluor-488 anti-mouse secondary antibody Cells were counterstained with DAPI. Cells were mounted onto slides using hydromount and examined under a Zeiss AxioPlan 2ie widefield microscope with an AxioCam HRm CCD camera. Images were analyzed using Image J software.

### Cell cycle analysis

Cell cycle analysis was carried out as previously detailed [30]. In brief, A375 melanoma cells were seeded in 24-well plates at a density of 4,600 cells per well. After 24 hours, the cells were treated with vehicle, 25, 50 and 100 µM of Leflunomide. After 72 hours, the cells were trypsinised and pelleted along with the culture medium. Cells were washed in PBS and fixed in ice-cold absolute ethanol. Cells were then stained with 200 µl PI/RNase A solution (Cell Signalling Technology). Cells were analyzed using a BD Accuri™ C6 flow cytometer (BD Biosciences) and the data was analyzed using the BD Accuri™ C6 Software and FlowJo (FLOWJO, LLC).

### Annexin V apoptosis assay

Apoptosis was assessed using an Annexin V-FITC Apoptosis detection kit FITC (eBioscience), according to the manufacturer’s instructions. Briefly, cells were seeded in 24-well plates at a density of 4,600 cells per well. After 24 hours, the cells were treated with vehicle or leflunomide (at concentrations of 25, 50 and 100 µM). After 72 hours cells were trypsinised, washed in PBS and treated with fluorochrome-conjugated Annexin V and propidium iodide as indicated in the protocol. Cells were analyzed on the BD Accuri™ C6 flow cytometer and the data analyzed using the instrument software.

### Western blot analysis

A375 melanoma cells were seeded and treated for 72 hours in varying drug conditions. Total protein extracts were then made using high SDS content lysis buffer (60 mM sucrose, 65 mM Tris-HCl, pH 6.8. 3% SDS). Protein concentration was determined using the *DC* protein assay (BIORAD). 10 μg of whole-cell protein lysate was loaded onto a 10% SDS-PAGE gel and transferred onto polyvinylidene difluoride membranes (Bio-Rad). A standard Western blot protocol was used for detection of the specific proteins as previously described [29].

Antibodies used included; Rabbit polyclonal phospho-ERK (1:1000, Cell Signalling Technology); Rabbit polyclonal phospho-p44/42 MAPK (1:1000, ERK1/2; Cell Signalling Technology); Mouse monoclonal HSC-70 (1:1000, Santa-Cruz Biotechnology); Rabbit polyclonal Mcl-1 (1:500, Santa-Cruz Biotechnology); Rabbit polyclonal anti-BIM (1:500, Merck-Millipore); Rabbit polyclonal PARP (1:1000, Cell Signalling Technology); Rabbit polyclonal PUMA (1:1000, Cell Signalling Technology); anti-rabbit IgG HRP linked secondary antibody (1:2000, Jackson ImmunoResearch); Anti-mouse IgG HRP linked secondary antibody (1:2000, Jackson ImmunoResearch); All anti-rabbit IgG HRP linked secondary antibody (1:2000, Cell Signalling Technology).

### Mouse xenograft study

8–10-week-old female severe immunodeficiency (SCID) mice were purchased from Charles River Laboratories. All procedures were performed under UK Home Office approved protocols and the University of East Anglia local guidelines. Experiments were conducted strictly in accordance with the locally approved animal handling protocol.

A total of 3 × 10^6^ M375 melanoma cells were injected subcutaneously into 40 SCID mice. After approximately 4 weeks when the tumors were palpable, the mice were randomized into 4 arms. The 4 arms were; vehicle alone, leflunomide alone, selumetinib alone and leflunomide and selumetinib in combination. There were 10 mice in each arm. The drug regime was administered for 12 days. Leflunomide was administered by intraperitoneal (IP) injection daily at 7.5 mg/kg. Selumetinib was administered by oral gavage (OG) twice daily at 30 mg/kg for the first two days and was then delivered once daily thereafter. The tumor volume was measured every three days with calipers. Tumor volume was measured by the formula 0.52 (length × width^2^). At the end of the experiment, the mice were culled and the excised tumors were weighed.

### Statistical analysis

Either one- or two-way ANOVA was used to analyze statistical significance of the data (as indicated in the figure legends), apart from *in vivo* tumor volume, where an unpaired student’s *t*-test was used. For all *in vitro* data, experiments were repeated a minimum of 3 times. The *P*-value was considered significant as follows; ^*^P < 0.05, ^**^P < 0.01, ^***^P < 0.001 and ^****^P < 0.0001.

For pharmacological analysis, IC_50_ values were generated using Prism Graphpad software (Graphpad Software, Inc.) and calculated using a nonlinear regression model. Combinatorial drug synergy was assessed by determination of combination index (CI), which was calculated using CalcuSyn (Biosoft) software using the median effects methods as described by Chou and Talalay [31, 32], CI values less than 0.7 indicated synergy, 0.7–0.9 weak synergy, 0.9–1.1 additivity, 1.1–1.45 indicated weak antagonism and greater than 1.45 antagonism.

## SUPPLEMENTARY MATERIALS FIGURES AND TABLES


