# Correction: The anti-rheumatic drug, leflunomide, synergizes with MEK inhibition to suppress melanoma growth

**DOI:** 10.18632/oncotarget.26415

**Published:** 2018-11-27

**Authors:** Kimberley Hanson, Stephen D. Robinson, Karamallah Al-Yousuf, Adam E. Hendry, Darren W. Sexton, Victoria Sherwood, Grant N. Wheeler

**Affiliations:** ^1^ School of Biological Sciences, University of East Anglia, Norwich Research Park, Norwich, NR4 7TJ, UK; ^2^ School of Pharmacy, University of East Anglia, Norwich Research Park, Norwich, NR4 7TJ, UK; ^3^ Present address: Division of Cancer Sciences, School of Medicine, Ninewells Hospital and Medical School, University of Dundee, Dundee, DD1 9SY, UK; ^4^ Norwich Medical School, University of East Anglia, Norwich Research Park, Norwich, NR4 7TJ, UK; ^5^ Present address: Pharmacy and Biomedical Sciences, Liverpool John Moores University, Liverpool, L3 3AF, UK

**This article has been corrected:** The correct Author name is given below:

**Stephen D. Robinson**

Original article: Oncotarget. 2018; 9:3815-3829. https://doi.org/10.18632/oncotarget.23378

