# Supplementary material for: The anti-rheumatic drug, leflunomide, synergizes with MEK inhibition to suppress melanoma growth
Source: Oncotarget. 2017 Dec 17;9(3):3815–29. doi: 10.18632/oncotarget.23378 (PMC5790502; doi:10.18632/oncotarget.23378)
Supplement: Supplementary file 1 [file oncotarget-09-3815-s001.pdf]

The anti-rheumatic drug, leflunomide, synergizes with MEK inhibition to suppress melanoma growth

SUPPLEMENTARY MATERIALS

Mitochondrial membrane potential [ΔΨM] assay

A375 melanoma cells were seeded in 12 well plates at a density of 10,000 cells per 1 ml of medium per well. After 24 hours, the cells were treated with vehicle or leflunomide. After 72 hours, the cells were incubated with 2 µM JC-1 (Life technologies) for 45 minutes at 37° C, 5% CO_2. Cells were then trypsinised, pelleted, washed in PBS and resuspended in 200 µl of fresh PBS before being analyzed on the BD Accuri™ C6 flow cytometer. Samples were exposed to 488 nm excitation, with JC-1 green monomer fluorescence detected at 533/30 nm in the FL1 channel and JC-1 red aggregates fluorescence detected at 585/20 nm in the FL2 channel. Signal compensation was performed to correct for spillover from FL1 into FL2. Data was analyzed using the BD Accuri™ C6 Software.

Mitotracker green staining

A375 melanoma cells were seeded in 12-well plates at a density of 10,000 cells per well. After 24 hours the cells were treated with vehicle or leflunomide at the indicated concentrations. After 72 hours, the cells were incubated with 25 nm Mitotracker green (Invitrogen) for 45 minutes at 37° C, 5% CO_2. Cells were trypsinised and pelleted, then washed in PBS and resuspended in fresh PBS before analysis on the BD Accuri™ C6 flow cytometer (with green fluorescence detected in the FL1 channel). Data was analyzed using the BD Accuri™ C6 Software.

REFERENCES

1. Tap WD, Gong KW, Dering J, Tseng Y, Ginther C, Pauletti G, Glaspy JA, Essner R, Bollag G, Hirth P, Zhang C, Slamon DJ. Pharmacodynamic characterization of the efficacy signals due to selective BRAF inhibition with PLX4032 in malignant melanoma. Neoplasia. 2010; 12:637–649.
2. Haluska FG, Tsao H, Wu H, Haluska FS, Lazar A, Goel V. Genetic alterations in signaling pathways in melanoma. Clin Cancer Res. 2006; 12:2301s–2307s.
3. von Euw E, Atefi M, Attar N, Chu C, Zachariah S, Burgess BL, Mok S, Ng C, Wong DJ, Chmielowski B, Lichter DI, Koya RC, McCannel TA, et al. Antitumor effects of the investigational selective MEK inhibitor TAK733 against cutaneous and uveal melanoma cell lines. Mol Cancer. 2012; 11:22.
4. Halaban R, Zhang W, Bacchiocchi A, Cheng E, Parisi F, Ariyan S, Krauthammer M, McCusker JP, Kluger Y, Sznol M. PLX4032, a selective BRAF(V600E) kinase inhibitor, activates the ERK pathway and enhances cell migration and proliferation of BRAF melanoma cells. Pigment Cell Melanoma Res. 2010; 23:190–200.
5. Panka DJ, Wang W, Atkins MB, Mier JW. The Raf inhibitor BAY 43-9006 (Sorafenib) induces caspase-independent apoptosis in melanoma cells. Cancer Res. 2006; 66:1611–1619.
6. Atefi M, Titz B, Tsoi J, Avramis E, Le A, Ng C, Lomova A, Lassen A, Friedman M, Chmielowski B, Ribas A, Graeber TG. CRAF R391W is a melanoma driver oncogene. Sci Rep. 2016; 6:27454.

Supplementary Table 1: The genetic status of frequently mutated melanoma genes in a selected human melanoma cell line panel

Cell Line	BRAF	NRAS	PTEN	PIK3CA	MITF (Amp)	MC1R
A375	V600E[1]	WT[2]	INTACT[1]	WT[1]	N[1]	R151C[1]
M202	WT[1]	Q61L[3]	INTACT[1]	U	N[1]	WT[1]
M229	V600E[3]	WT[1]	Del[1]	WT[1]	Y[3]	R151C[1]
M285	WT[3]	WT[3]	INTACT[4]	U	U	U
M296	WT[1]	Q61L[3]	INTACT[1]	WT[1]	N[1]	WT[1]
M375[¥]	WT[3]	WT[3]	INTACT[3]	WT[3]	U	U
SKmel5	V600E[1]	WT[1]	INTACT[1]	WT[5]	N[1]	V92M[1]
SKmel28	V600E[3]	WT[1]	Del[1]	WT[1]	Y[1]	L155T[1]

[¥]M375 cells harbour a *CRAF* R391W mutation[6].

Abbreviations: *BRAF*, v-raf murine sarcoma viral oncogene homolog B1; *NRAS*, Neuroblastoma RAS viral (v-ras) oncogene homolog; *PTEN*, Phosphatase and tensin homolog; *AKT*, v-akt murine thymoma viral oncogene homolog; *MITF*, Microphthalmia-associated transcription factor; *PIK3CA*, Phosphoinositide 3-Kinase catalytic subunit alpha; *MC1R*, Melanocortin receptor 1; Y, Yes; N, No; U, unknown; Amp, Amplification; WT, wild-type; Del, deleted.

Supplementary Tables 2-5: Summary tables for the combination index values for the A375, M229, M375 and M285 melanoma cell lines treated with leflunomide and selumetinib at the same time or with pre-treatment for 24 hours with leflunomide or selumetinib. In the tables, purple indicates antagonism, orange indicates additive and green indicates synergism

Supplementary Table 2:

A375 melanoma cell line				
		Combination index		
**Selumetinib (µM)**	**Leflunomide (µM)**	**Leflunomide and selumetinib at the same time**	**Leflunomide 24 hour pre treatment**	**Selumetinib 24 hour pre treatment**
0.025	12.5	1.621	0.88363	0.9167
0.05	12.5	1.526	1.19666	1.22762
0.1	12.5	1.561	1.41595	1.26827
0.025	25	1.59	1.22344	1.10714
0.05	25	1.699	1.28659	1.16125
0.1	25	1.814	1.55264	1.45044
0.025	50	1.414	1.27643	1.06051
0.05	50	1.507	1.20974	1.13169
0.1	50	1.646	1.6285	1.42372

Supplementary Table 3:

M229 melanoma cell line				
		Combination index		
**Selumetinib (µM)**	**Leflunomide (µM)**	**Leflunomide and selumetinib at the same time**	**Leflunomide 24 hour pre treatment**	**Selumetinib 24 hour pre treatment**
0.025	12.5	0.827	2.1567	0.94701
0.05	12.5	0.743	2.41606	0.84505
0.1	12.5	0.939	2.53868	1.06029
0.025	25	1.011	1.52786	1.28151
0.05	25	0.831	1.70506	1.11133
0.1	25	0.94	2.37912	0.77127
0.025	50	0.801	1.17593	1.08006
0.05	50	0.617	1.32693	0.92337
0.1	50	0.846	1.30465	0.59609

Supplementary Table 4:

M375 melanoma cell line				
		Combination index		
Selumetinib (µM)	Leflunomide (µM)	Leflunomide and selumetinib at the same time	Leflunomide 24 hour pre treatment	Selumetinib 24 hour pre treatment
0.025	12.5	0.528	1.54708	0.98374
0.05	12.5	0.661	2.34111	1.16188
0.1	12.5	0.834	2.83633	1.9737
0.025	25	0.473	1.94343	1.04623
0.05	25	0.549	2.06715	1.38204
0.1	25	0.754	2.56872	1.85773
0.025	50	0.328	1.04531	0.99408
0.05	50	0.327	1.06653	1.03813
0.1	50	0.519	1.3012	0.95124

Supplementary Table 5:

M285 melanoma cell line				
		Combination index		
Selumetinib (µM)	Leflunomide (µM)	Leflunomide and selumetinib at the same time	Leflunomide 24 hour pre treatment	Selumetinib 24 hour pre treatment
0.025	12.5	0.704	0.65261	0.34513
0.05	12.5	0.95	0.82107	0.62615
0.1	12.5	1.033	0.60839	0.67090
0.025	25	0.657	0.54502	0.46565
0.05	25	0.709	0.50348	0.51353
0.1	25	0.777	0.43781	0.79389
0.025	50	0.568	0.52379	0.33812
0.05	50	0.665	0.38382	0.34852
0.1	50	0.515	0.25503	0.45099

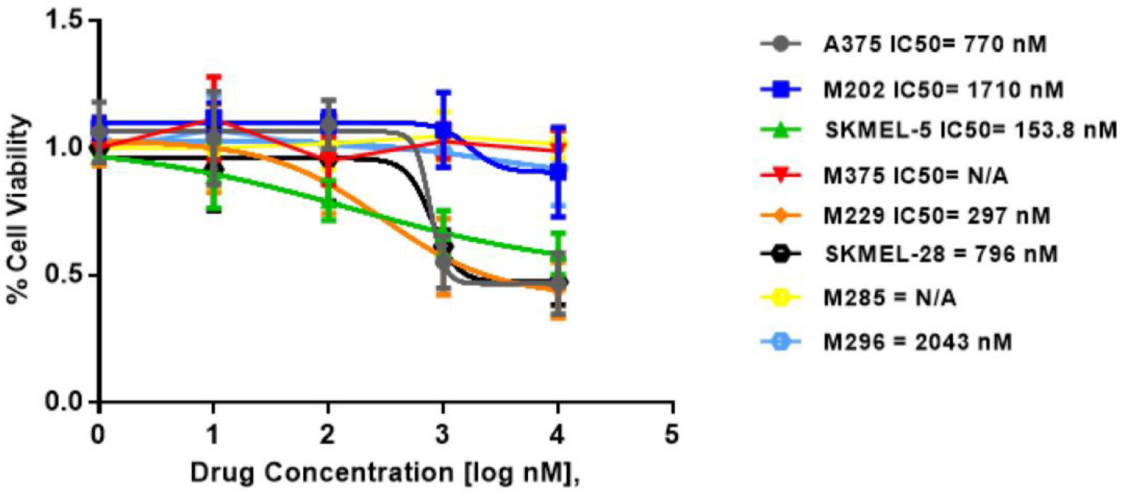

Supplementary Figure 1: Vemurafenib causes a dose dependent decrease in cell viability in *BRAF*^{V600E} mutant cell lines but not wild-type *BRAF* cells. Wild-type cell lines; M202 (dark blue), M285 (yellow), M375 (red) and M296 (light blue). *BRAF*^{V600E} mutant cell lines; A375 (khaki), M229 (orange), SKmel28 (black) and SKmel5 (green). Cell viability was determined by using CellTiter-Glo reagent and all values are represented as a percentage (%) relative to the vehicle control. Data is presented as the mean ± SEM of three independent experiments each performed with cell culture triplicates. The IC_{50} for each cell line is shown in Table 1.

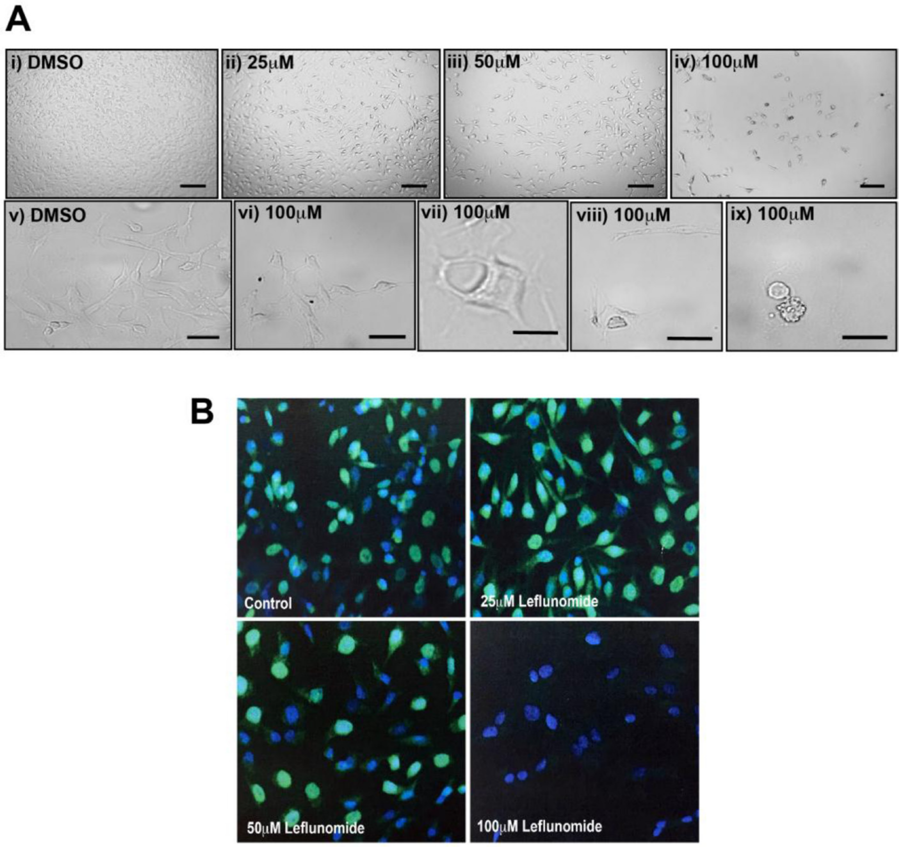

Supplementary Figure 2: Dose-dependent effect of leflunomide on A375 monolayers. (A) Phenotypic images of A375 cells treated with leflunomide for 72 hours. Images i–iv were taken at 10× magnification, whilst images v–ix were taken at 40× magnification. (**B**) Representative images of A375 cells labelled with BrdU after treatment for 72 hours with increasing concentrations of Leflunomide.

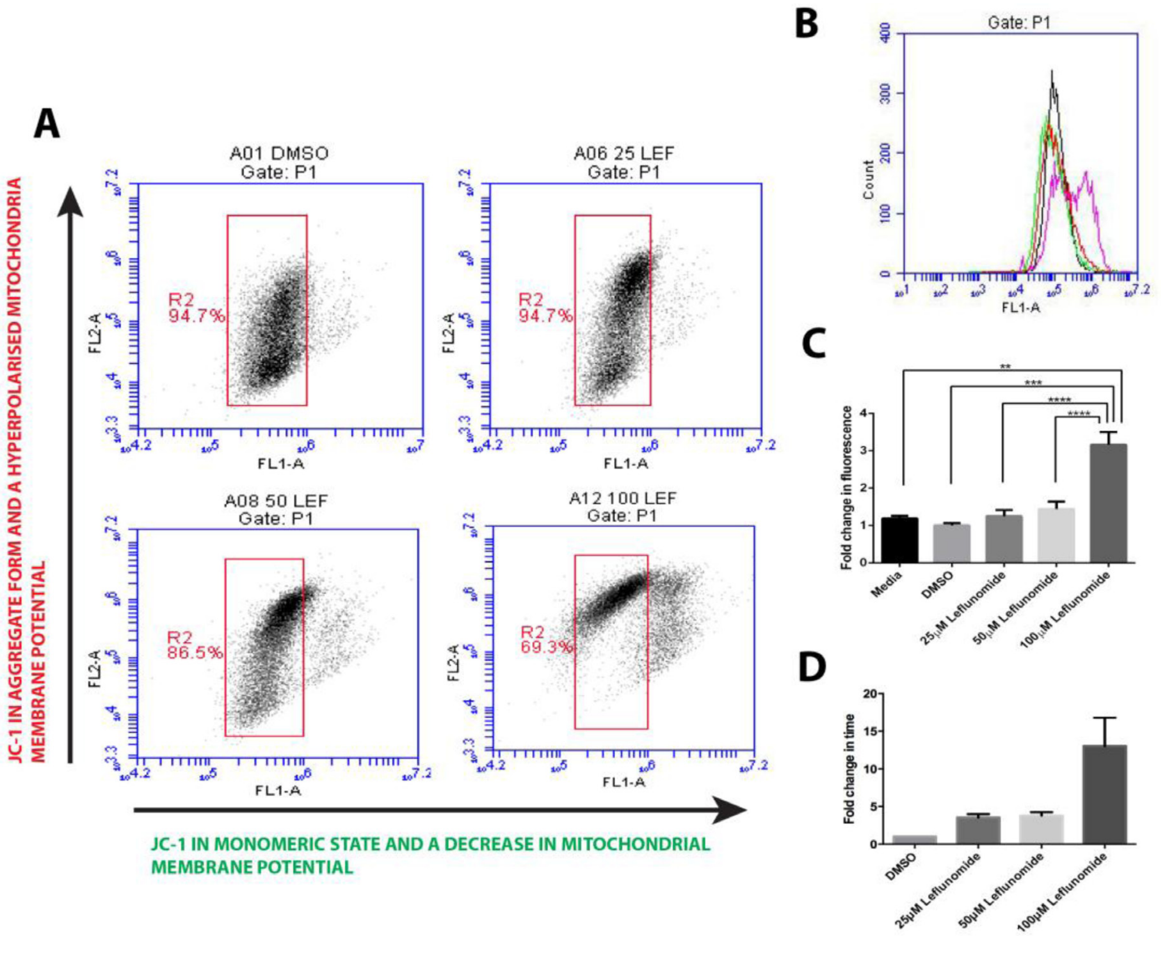

Supplementary Figure 3: Leflunomide affects mitochondrial activity. (A) Representative JC-1 plots measuring mitochondrial membrane potential determined by flow cytometry. A375 cells were treated with DMSO, 25, 50 and 100 μM leflunomide for 72 hours and stained with JC-1. (**B**) Representative data plot showing the intensity of green fluorescence for DMSO, 25, 50 and 100 μM leflunomide treated A375 cells. DMSO treated cells are shown in black, 25, 50 and 100 μM leflunomide treated cells are shown in green, red and magenta, respectively. (**C**) Quantification of the fold change of the intensity of green fluorescence between 25, 50 and 100 μM leflunomide treated A375 cells and DMSO control cells. Data is presented as the mean ± SEM of three independent experiments each performed with cell culture triplicate. Asterisks indicate the degree of statistical difference comparing each leflunomide condition to the DMSO control determined by one-way ANOVA with Turkey's post-hoc test. $^{***}P \leq 0.001$ and $^{****}P \leq 0.0001$. (**D**) Increasing concentrations of leflunomide cause an increase in the fold change in sample acquisition time compared to the DMSO control (indicative of sample cell density). Data is presented as the mean ± SEM of three independent experiments each performed with cell culture triplicate.

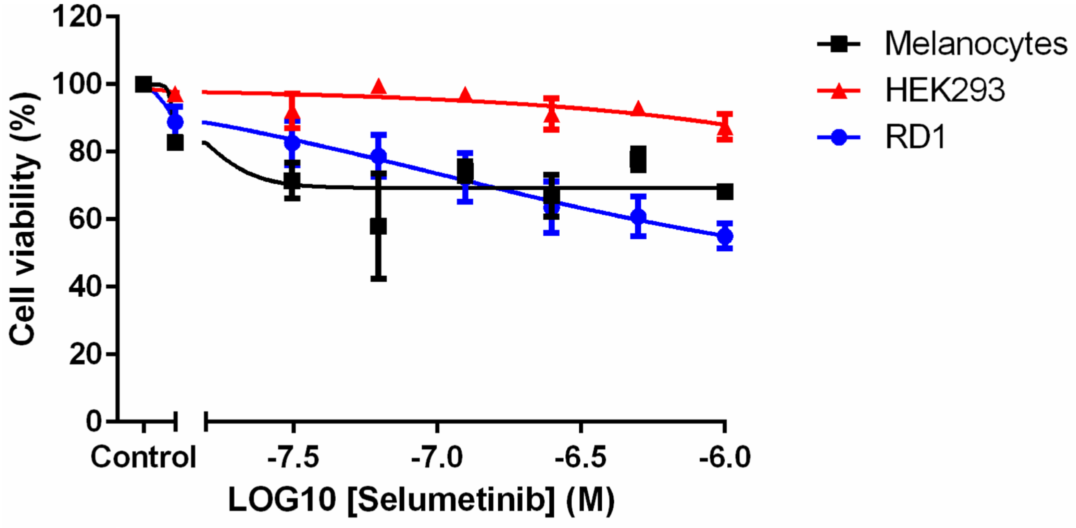

Supplementary Figure 4: Selumetinib does not affect the cell viability of non-melanoma cell lines. Selumetinib had little effect on cell viability of the melanocytes (shown in black), HEK293 (shown in red) and RD1 (shown in blue) cell lines. Cell viability was determined by using CellTiter-Glo reagent and all values are represented as a percentage (%) relative to the vehicle control. Data is presented as the mean ± SEM of three independent experiments each performed with cell culture triplicate.

Supplementary Figure 5: Leflunomide and Selumetinib synergize in melanoma cells.
Extra graphs from Synergy experiments

- All cell viability graphs of combinatorial studies (8 melanoma cell lines).
- All combination index graphs (8 melanoma cell lines).

A

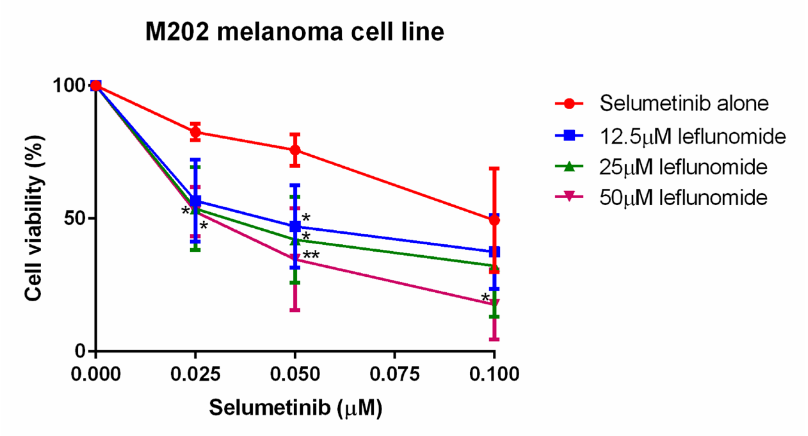

B

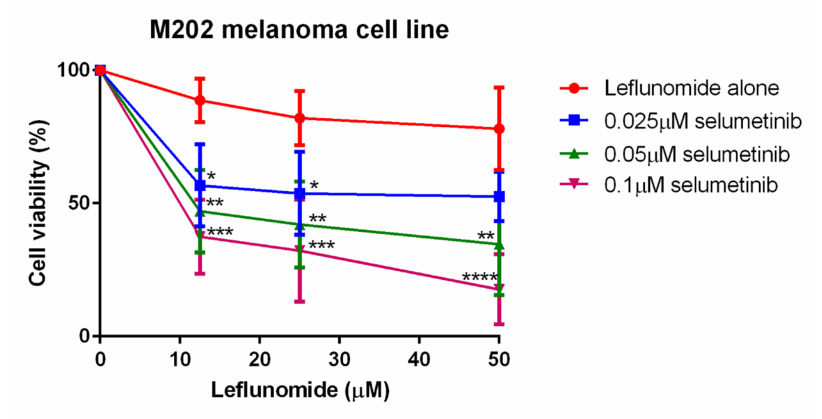

Treatment condition	IC_{50} (μM)
Meki alone	0.092027
Mek + Lef 12.5	0.05853
Mek + Lef 25	0.04943
Mek + Lef 50	0.039633

Treatment condition	IC_{50} (μM)
Leflunomide alone	51.183
Leflunomide + Meki 0.025	54.6125
Leflunomide + Meki 0.05	34.2943
Leflunomide + Meki 0.1	16.2383

Supplementary Figure 5-1: M202. The combination of leflunomide and selumetinib reduces cell viability in the M202 cell line. Graph (**A**) shows the concentrations of selumetinib along the x-axis. The statistical analysis on this graph compares the combinations of drugs to selumetinib alone. Graph (**B**) shows the concentrations of leflunomide along the x-axis. The statistical analysis on this graph compares the drug combinations to leflunomide alone. The IC_{50}'s are for each graph are also shown. Cell viability was determined by using CellTiter-Glo reagent and all values are represented as a percentage (%) relative to the vehicle control. Data is presented as the mean ± SEM of three independent experiments each performed with cell culture triplicate. Asterisks indicate the degree of statistical difference comparing each leflunomide and selumetinib condition to leflunomide alone (graph A) or selumetinib alone (graph B). Statistical analysis was determined by two-way ANOVA with Turkey's post-hoc test. $^{*}P \leq 0.05$, $^{**}P \leq 0.01$, $^{***}P \leq 0.001$ and $^{****}P \leq 0.0001$.

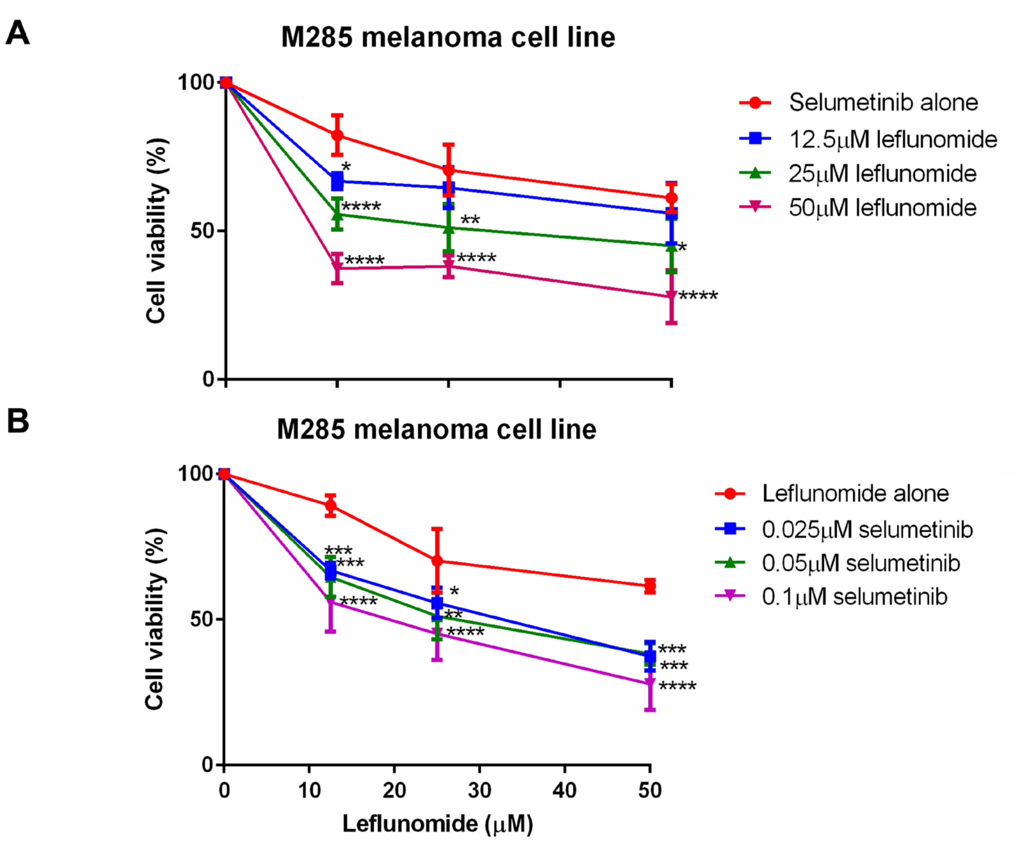

Treatment condition	IC_{50} (μM)
Meki alone	0.165867
Mek + Lef 12.5	0.175397
Mek + Lef 25	0.06337
Mek + Lef 50	0.02238

Treatment condition	IC_{50} (μM)
Leflunomide alone	72.3867
Leflunomide + Meki 0.025	29.57
Leflunomide + Meki 0.05	26.9967
Leflunomide + Meki 0.1	17.7733

Supplementary Figure 5-2: M285. The combination of leflunomide and selumetinib reduced cell viability in the M285 cell line. Graph (**A**) shows the concentrations of selumetinib along the x-axis. The statistical analysis on this graph compared the combinations of drugs to selumetinib alone. Graph (**B**) shows the concentrations of leflunomide along the x-axis. The statistical analysis on this graph compared the drug combinations to leflunomide alone. IC_{50}'s for each graph are also shown. Cell viability was determined by using CellTiter-Glo reagent and all values are represented as a percentage (%) relative to the vehicle control. Data is presented as the mean ± SEM of three independent experiments each performed with cell culture triplicate. Asterisks indicate the degree of statistical difference comparing each leflunomide and selumetinib condition to leflunomide alone (graph A) or selumetinib alone (graph B). Statistical analysis was determined by two-way ANOVA with Turkey's post-hoc test. $^{*}P \leq 0.05$, $^{**}P \leq 0.01$, $^{***}P \leq 0.001$ and $^{****}P \leq 0.0001$.

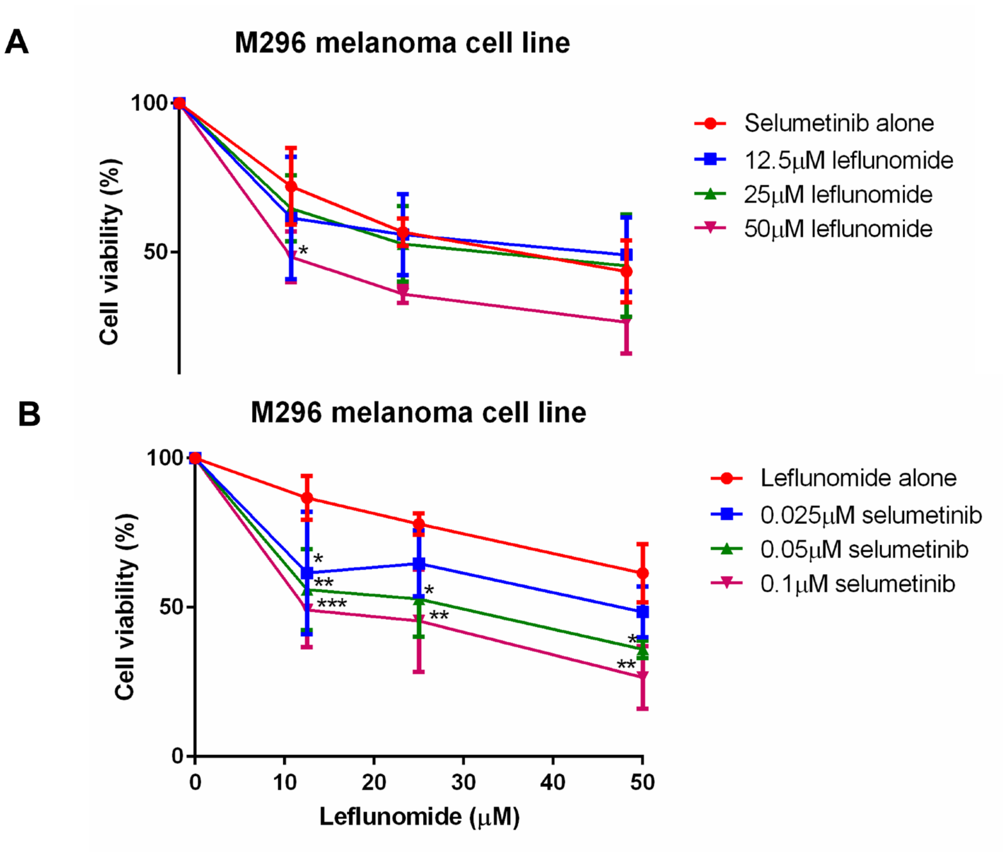

Treatment condition	IC_{50} (μM)
Meki alone	0.073586
Mek + Lef 12.5	0.0845
Mek + Lef 25	0.10596
Mek + Lef 50	0.02805

Treatment condition	IC_{50} (μM)
Leflunomide alone	109.936
Leflunomide + Meki 0.025	42.56
Leflunomide + Meki 0.05	21.3153
Leflunomide + Meki 0.1	17.3686

Supplementary Figure 5-3: M296. The combination of leflunomide and selumetinib reduced cell viability in the M296 cell line. Graph (**A**) shows the concentrations of selumetinib along the x-axis. The statistical analysis on this graph compared the combinations of drugs to selumetinib alone. Graph (**B**) shows the concentrations of leflunomide along the x-axis. The statistical analysis on this graph compared the drug combinations to leflunomide alone. The IC_{50}'s for each graph are also shown. Cell viability was determined by using CellTiter-Glo reagent and all values are represented as a percentage (%) relative to the vehicle control. Data is presented as the mean ± SEM of three independent experiments each performed with cell culture triplicate. Asterisks indicate the degree of statistical difference comparing each leflunomide and selumetinib condition to leflunomide alone (graph A) or selumetinib alone (graph B). Statistical analysis was determined by two-way ANOVA with Turkey's post-hoc test. $^{*}P \leq 0.05$, $^{**}P \leq 0.01$, $^{***}P \leq 0.001$ and $^{****}P \leq 0.0001$.

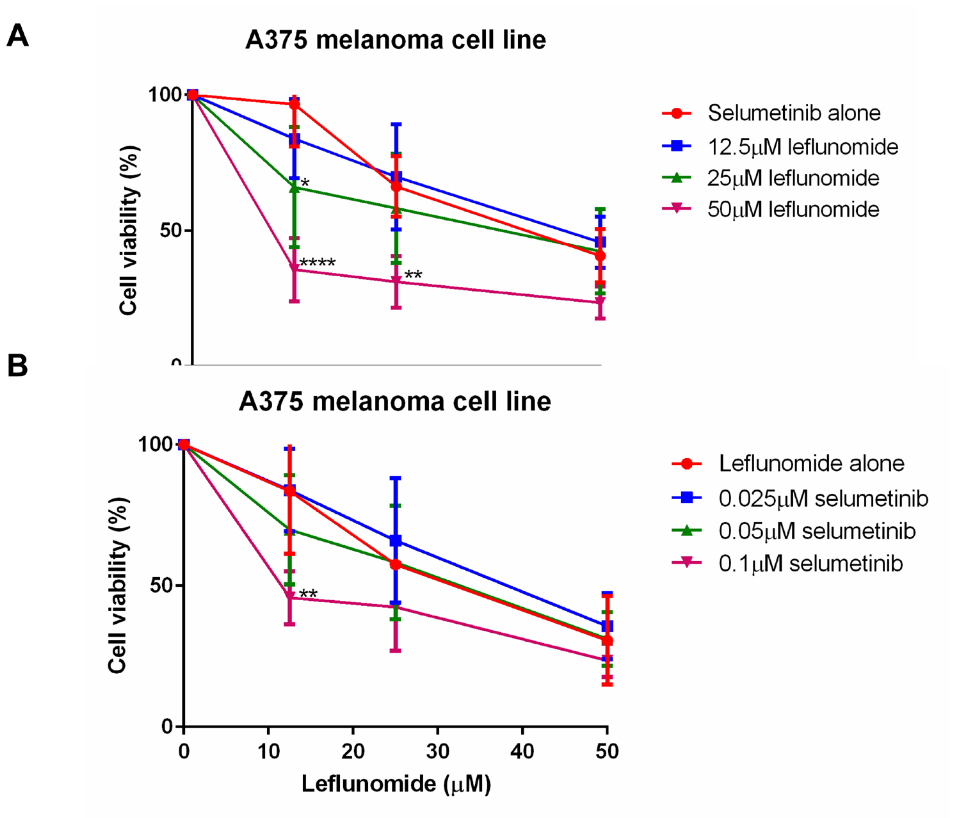

Treatment condition	IC_{50} (μM)
**Meki alone**	0.085327
**Mek + Lef 12.5**	0.083253
**Mek + Lef 25**	0.070023
**Mek + Lef 50**	0.019527

Treatment condition	IC_{50} (μM)
**Leflunomide alone**	31.8067
**Leflunomide + Meki 0.025**	34.85
**Leflunomide + Meki 0.05**	27.53
**Leflunomide + Meki 0.1**	13.0997

Supplementary Figure 5-4: A375. The combination of leflunomide and selumetinib reduced cell viability in the A375 cell line. Graph (**A**) shows the concentrations of selumetinib along the x-axis. The statistical analysis on this graph compared the combinations of drugs to selumetinib alone. Graph (**B**) shows the concentrations of leflunomide along the x-axis. The statistical analysis on this graph compared the drug combinations to leflunomide alone. The IC_{50}'s for each graph are also shown. Cell viability was determined by using CellTiter-Glo reagent and all values are represented as a percentage (%) relative to the vehicle control. Data is presented as the mean ± SEM of three independent experiments each performed with cell culture triplicate. Asterisks indicate the degree of statistical difference comparing each leflunomide and selumetinib condition to leflunomide alone (graph A) or selumetinib alone (graph B). Statistical analysis was determined by two-way ANOVA with Turkey's post-hoc test. $^{*}P \leq 0.05$, $^{**}P \leq 0.01$, $^{***}P \leq 0.001$ and $^{****}P \leq 0.0001$.

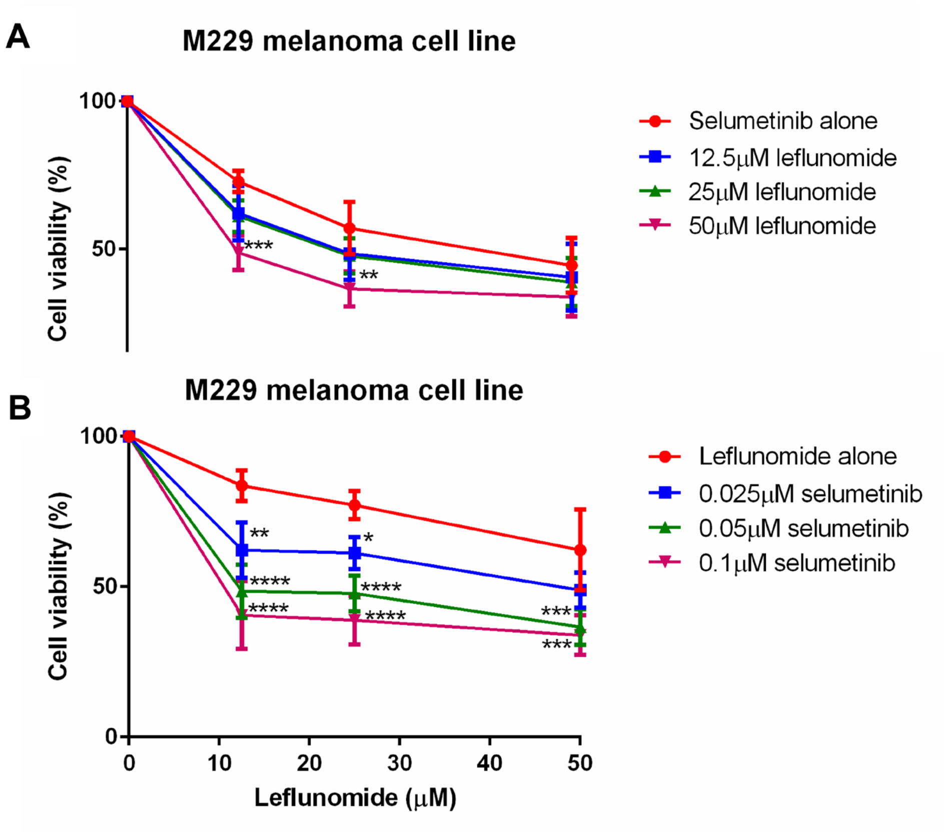

Treatment condition	IC_{50} (μM)
Meki alone	0.082073
Mek + Lef 12.5	0.058577
Mek + Lef 25	0.050353
Mek + Lef 50	0.030117

Treatment condition	IC_{50} (μM)
Leflunomide alone	89.115
Leflunomide + Meki 0.025	53.16
Leflunomide + Meki 0.05	15.525
Leflunomide + Meki 0.1	8.773667

Supplementary Figure 5-5: M229. The combination of leflunomide and selumetinib reduced cell viability in the M229 cell line. Graph (**A**) shows the concentrations of selumetinib along the x-axis. The statistical analysis on this graph compared the combinations of drugs to selumetinib alone. Graph (**B**) shows the concentrations of leflunomide along the x-axis. The statistical analysis on this graph compared the drug combinations to leflunomide alone. The IC_{50}'s for each graph are also shown. Cell viability was determined by using CellTiter-Glo reagent and all values are represented as a percentage (%) relative to the vehicle control. Data is presented as the mean ± SEM of three independent experiments each performed with cell culture triplicate. Asterisks indicate the degree of statistical difference comparing each leflunomide and selumetinib condition to leflunomide alone (graph A) or selumetinib alone (graph B). Statistical analysis was determined by two-way ANOVA with Turkey's post-hoc test. $^{*}P \leq 0.05$, $^{**}P \leq 0.01$, $^{***}P \leq 0.001$ and $^{****}P \leq 0.0001$.

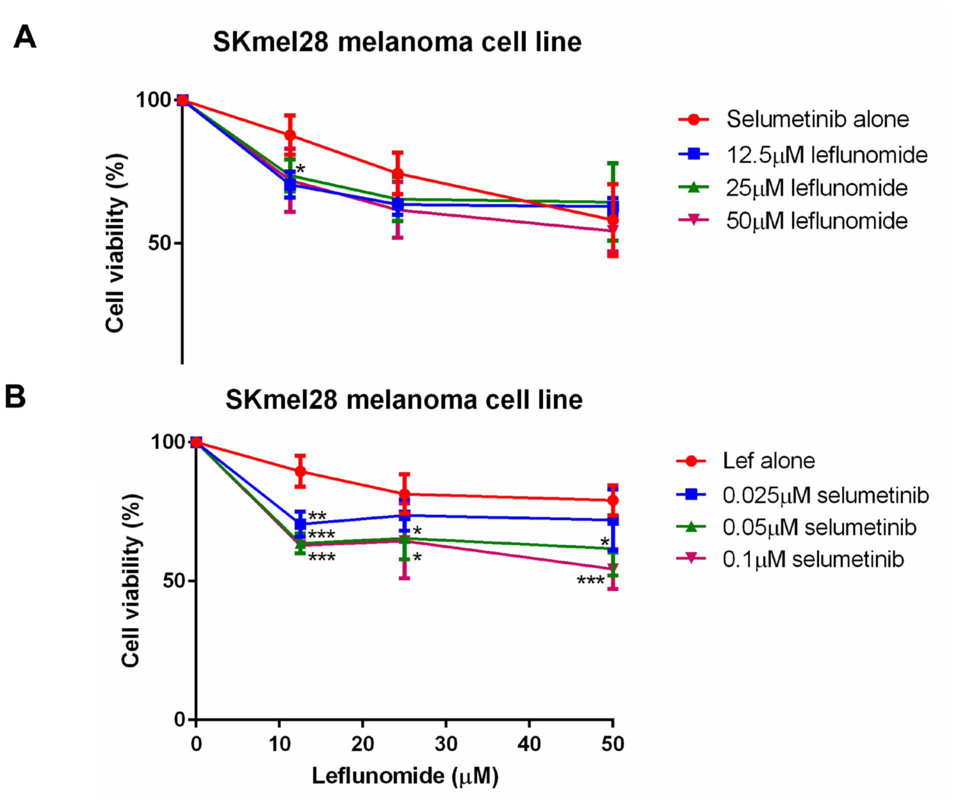

Treatment condition	IC_{50} (μM)
**Meki alone**	0.1684
**Mek + Lef 12.5**	0.1875
**Mek + Lef 25**	0.585
**Mek + Lef 50**	0.112

Treatment condition	IC_{50} (μM)
**Leflunomide alone**	n/a
**Leflunomide + Meki 0.025**	n/a
**Leflunomide + Meki 0.05**	n/a
**Leflunomide + Meki 0.1**	n/a

Supplementary Figure 5-6: SKmel28. The combination of leflunomide and selumetinib reduced cell viability in the SKmel28 cell line. Graph (**A**) shows the concentrations of selumetinib along the x-axis. The statistical analysis on this graph compared the combinations of drugs to selumetinib alone. Graph (**B**) shows the concentrations of leflunomide along the x-axis. The statistical analysis on this graph compared the drug combinations to leflunomide alone. The IC_{50}'s for each graph are also shown. Cell viability was determined by using CellTiter-Glo reagent and all values are represented as a percentage (%) relative to the vehicle control. Data is presented as the mean ± SEM of three independent experiments each performed with cell culture triplicate. Asterisks indicate the degree of statistical difference comparing each leflunomide and selumetinib condition to leflunomide alone (graph A) or selumetinib alone (graph B). Statistical analysis was determined by two-way ANOVA with Turkey's post-hoc test. $^{*}P \leq 0.05$, $^{**}P \leq 0.01$, $^{***}P \leq 0.001$ and $^{****}P \leq 0.0001$.

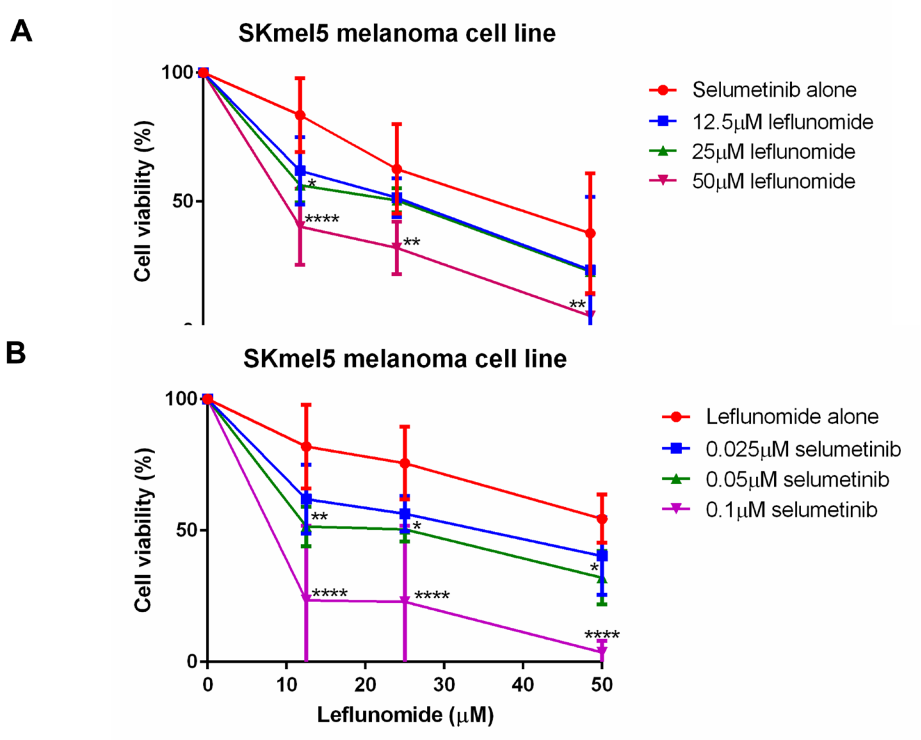

Treatment condition	IC_{50} (μM)
**Meki alone**	0.088842
**Mek + Lef 12.5**	0.052304
**Mek + Lef 25**	0.048602
**Mek + Lef 50**	0.022102

Treatment condition	IC_{50} (μM)
**Leflunomide alone**	62.152
**Leflunomide + Meki 0.025**	31.5225
**Leflunomide + Meki 0.05**	18.852
**Leflunomide + Meki 0.1**	8.2473

Supplementary Figure 5-7: SKmel5. The combination of leflunomide and selumetinib reduced cell viability in the SKmel5 cell line. Graph (**A**) shows the concentrations of selumetinib along the x-axis. The statistical analysis on this graph compared the combinations of drugs to selumetinib alone. Graph (**B**) shows the concentrations of leflunomide along the x-axis. The statistical analysis on this graph compared the drug combinations to leflunomide alone. The IC_{50}'s for each graph are also shown. Cell viability was determined by using CellTiter-Glo reagent and all values are represented as a percentage (%) relative to the vehicle control. Data is presented as the mean ± SEM of three independent experiments each performed with cell culture triplicate. Asterisks indicate the degree of statistical difference comparing each leflunomide and selumetinib condition to leflunomide alone (graph A) or selumetinib alone (graph B). Statistical analysis was determined by two-way ANOVA with Turkey's post-hoc test. $^{*}P \leq 0.05$, $^{**}P \leq 0.01$, $^{***}P \leq 0.001$ and $^{****}P \leq 0.0001$.

A

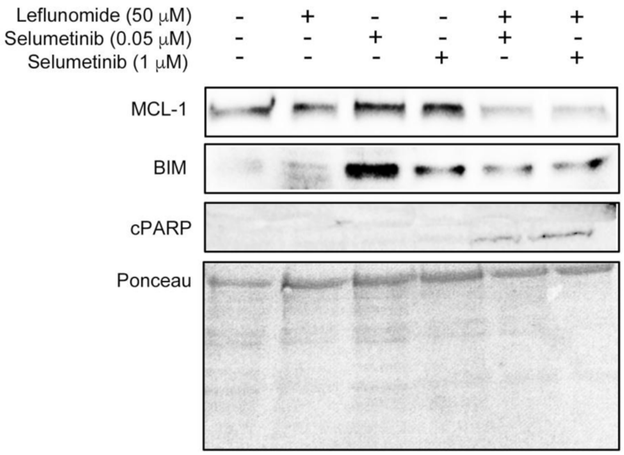

B

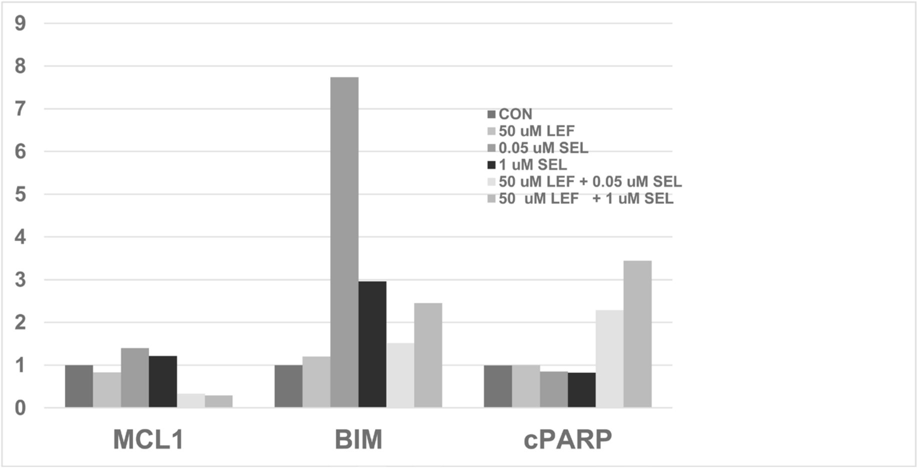

Supplementary Figure 6: Apoptotic markers in melanoma cells in response to leflunomide and selumetinib treatment. (A) Western blot analysis detecting the presence for anti- (MCL-1) and pro-apoptotic markers (BIM and cleaved PARP; cPARP) in A375 melanoma cells. Cells were subject to treatment with leflunomide and selumetinib alone and in combination at the indicated concentrations. 10 µg of protein was loaded into each lane. (**B**) Densitometry plot of western blot shown in A.

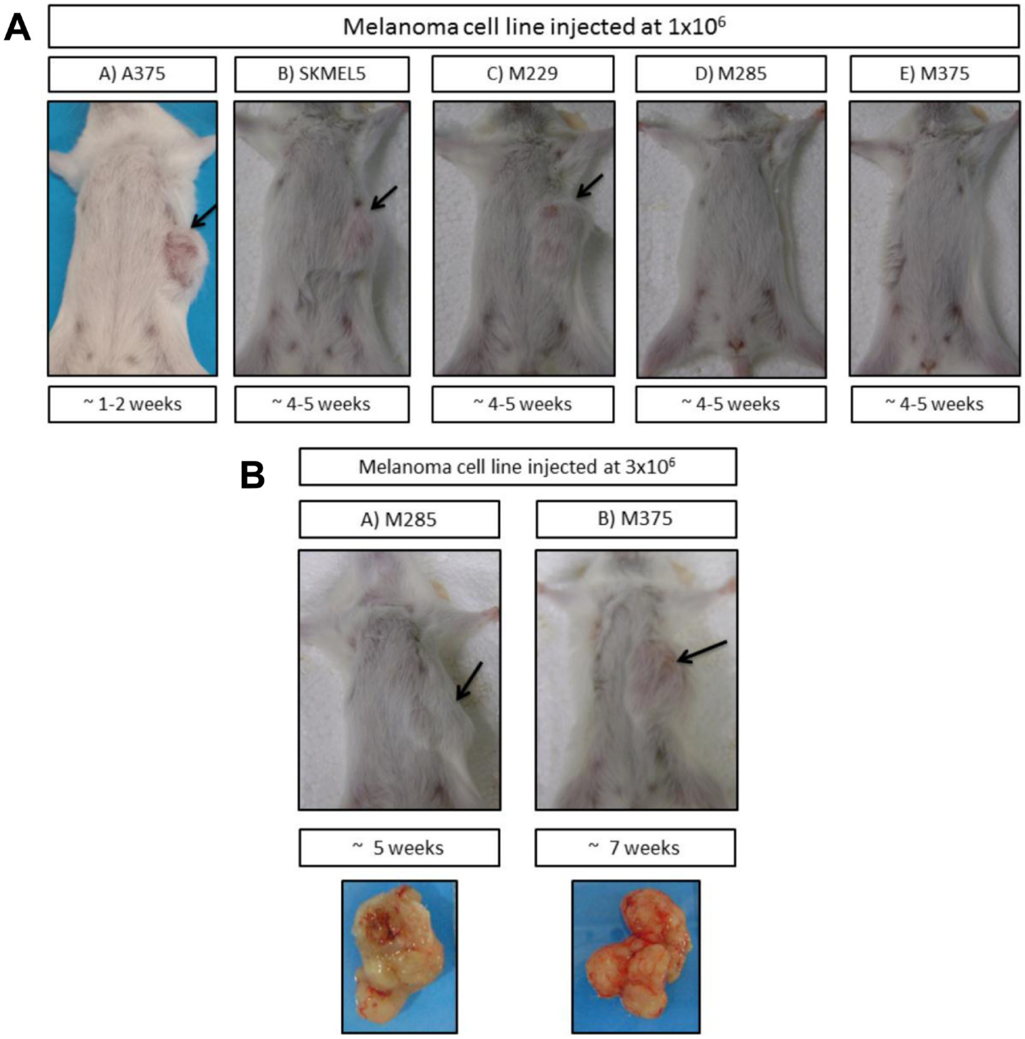

Supplementary Figure 7: SCID mouse xenografts with human melanoma cells. (**A**) Results showing successful tumor engraftment for the A375, SKmel5 and M229 cell lines in the SCID mouse strain, but not for the M285 and M375 melanoma cell lines. In each case 1×10^6 were injected subcutaneously. Black arrows indicate the location of the tumors. (**B**) Results of the repeated pilot study showing tumors for the M285, and M375 cell lines. In each case 3×10^6 cells were injected. Black arrows indicate the location of the tumors. Excised tumors shown in lower panel.
